# Viromes of Ten Alfalfa Plants in Australia Reveal Diverse Known Viruses and a Novel RNA Virus

**DOI:** 10.3390/pathogens9030214

**Published:** 2020-03-13

**Authors:** Samira Samarfard, Alistair R. McTaggart, Murray Sharman, Nicolás E. Bejerman, Ralf G. Dietzgen

**Affiliations:** 1 Queensland Alliance for Agriculture and Food Innovation, The University of Queensland, St Lucia, Queensland 4072, Australia; s.samarfard@uq.edu.au; 2 Queensland Alliance for Agriculture and Food Innovation, The University of Queensland, Ecosciences Precinct, Dutton Park, Queensland 4102, Australia; a.mctaggart@uq.edu.au; 3 Department of Agriculture and Fisheries, Ecosciences Precinct, Dutton Park, Queensland 4102, Australia; murray.sharman@daf.qld.gov.au; 4 Instituto de Patología Vegetal–Centro de Investigaciones Agropecuarias–Instituto Nacional de Tecnología Agropecuaria (IPAVE-CIAP-INTA), Córdoba 5020, Argentina; nicobejerman@gmail.com; 5 Consejo Nacional de Investigaciones Científicas y Técnicas (CONICET), Unidad de Fitopatología y Modelización Agrícola, Córdoba 5020, Argentina

**Keywords:** high throughput sequencing, alfalfa viruses, emaravirus, mastrevirus, double-stranded RNA

## Abstract

Alfalfa plants in the field can display a range of virus-like symptoms, especially when grown over many years for seed production. Most known alfalfa viruses have RNA genomes, some of which can be detected using diagnostic assays, but many viruses of alfalfa are not well characterized. This study aims to identify the RNA and DNA virus complexes associated with alfalfa plants in Australia. To maximize the detection of RNA viruses, we purified double-stranded RNA (dsRNA) for high throughput sequencing and characterized the viromes of ten alfalfa samples that showed diverse virus-like symptoms. Using Illumina sequencing of tagged cDNA libraries from immune-captured dsRNA, we identified sequences of the single-stranded RNA viruses, alfalfa mosaic virus (AMV), bean leafroll virus, a new emaravirus tentatively named alfalfa ringspot-associated virus, and persistent dsRNA viruses belonging to the families *Amalgaviridae* and *Partitiviridae*. Furthermore, rolling circle amplification and restriction enzyme digestion revealed the complete genome of chickpea chlorosis Australia virus, a mastrevirus (family *Geminiviridae*) previously reported only from chickpea and French bean that was 97% identical to the chickpea isolate. The sequence data also enabled the assembly of the first complete genome (RNAs 1–3) of an Australian AMV isolate from alfalfa.

## 1. Introduction

Alfalfa or lucerne (*Medicago sativa* L.) is a legume cultivated in more than 80 countries on about 45 million ha and used as silage for grazing livestock [1,2]. Virus diseases can affect production of alfalfa hay and seed [3]. A large and increasing number of viruses is known to infect alfalfa globally. Surveys of alfalfa using serological assays showed that alfalfa mosaic virus (AMV; genus *Alfamovirus*, family *Bromoviridae*), bean leafroll virus (BLRV; genus *Luteovirus*, family *Luteoviridae*), and cucumber mosaic virus (CMV; genus *Cucumovirus*, family *Bromoviridae*) are the most frequently detected viruses in Australian alfalfa pastures [3,4,5,6].

In recent years, several novel viruses have been identified in alfalfa worldwide, with the help of high throughput sequencing (HTS). For example, in 2010, alfalfa dwarf disease (ADD), which affects hay yield and quality of alfalfa, was reported from Argentina [7]. HTS of ADD-diseased plants revealed the presence of several RNA viruses, AMV, BLRV, alfalfa dwarf virus (ADV; genus *Cytorhabdovirus*, family *Rhabdoviridae*) and alfalfa enamovirus 1 (genus *Enamovirus*, family *Luteoviridae*), and the DNA virus alfalfa leaf curl virus (ALCV; genus *Capulavirus*, family *Geminiviridae*) [8,9,10,11,12]. More recently, alfalfa-associated nucleorhabdovirus (genus *Nucleorhabdovirus*, family *Rhabdoviridae*), alfalfa virus F (genus *Marafivirus*, family *Tymoviridae*), alfalfa virus S (genus *Allexivirus*, family *Alphaflexiviridae*), *Medicago sativa* deltapartitivirus (genus *Deltpartitivirus*, family *Partitiviridae*), and alphapartitiviruses including *Medicago sativa* alphapatitivirus 1 and 2 (family *Partitiviridae*) were identified in alfalfa with HTS [13,14,15,16,17,18]. 

Non-targeted HTS is a broad-spectrum diagnostic screening tool with high sensitivity to detect even low-titre viruses. It has many advantages over serological and molecular detection methods that require specific antisera or prior knowledge of nucleotide sequences [19]. Metagenomic HTS has significantly influenced the diagnosis of known and unknown plant viruses as well as genome diversity and evolutionary investigations at different taxonomic levels [14,15,16,17,18,19,20,21,22,23,24,25,26]. Recent advances in HTS technology and improved bioinformatics pipelines have appreciably reduced sequencing costs and time.

HTS approaches to identify viruses and viroids mainly differ in (1) plant sample preparation by enriching various types of genetic materials including messenger RNA (mRNA), total RNA with or without ribosomal RNA depletion, nucleic acids extracted from purified virions, small RNAs, and double-stranded (ds) RNAs; (2) sequencing platforms; and (3) bioinformatics used for assembly of contigs and viral genomes [27,28,29,30,31,32]. 

Sequencing total plant RNA generates more than 90% host transcript data, which is a challenge for detection of low-titre viruses [33]. The abundance of viral sequence reads depends on the virus titre and the efficiency of ribosomal RNA removal. Enrichment of viral nucleic acids prior to sequencing library preparation has been shown to enhance sensitivity of viral sequence detection [20,34,35]. Sequencing enriched mRNAs may not optimally represent viruses that lack terminal poly(A) sequences. Specific protocols for virus purification are required to sequence viral genomes from enriched virions [36]. 

Both DNA and RNA viruses can be detected by sequencing of small RNAs generated by RNA silencing during plant defence [36,37,38]. However, presence of small endogenous plant RNAs can interfere with viral genome assembly and coverage of the viral genome can be uneven when sequenced from small RNAs [39,40]. Since plants generally do not generate high molecular weight dsRNAs, the presence of such dsRNAs is mainly attributed to the presence of replicating ssRNA or to dsRNA viruses. Therefore, enriching for dsRNAs will lead to a greater percentage of sequence reads from viruses and improved detection sensitivity [41,42,43,44]. However, DNA viruses do not produce dsRNA and detection of these viruses requires alternative enrichment methods [27]. 

In this study, we used metagenomics with Illumina sequencing of tagged cDNA libraries derived from immuno-captured dsRNA to profile the RNA viromes of ten alfalfa samples that showed diverse virus-like symptoms. We specifically sought to identify the biodiversity of alfalfa RNA viruses and to characterize viral taxa that may be new to Australia. We also investigated the possible presence of novel DNA viruses circulating within Australian alfalfa pastures, which may pose a biosecurity threat for seed and hay production. DNA viruses were detected by Sanger sequencing of cloned rolling circle amplified DNA from selected alfalfa samples with leaf curl/roll-like symptoms that had tested negative for ALCV [45].

## 2. Results

### 2.1. Identification and Taxonomic Profiling of Viruses in Australian Alfalfa

**Figure 1:**
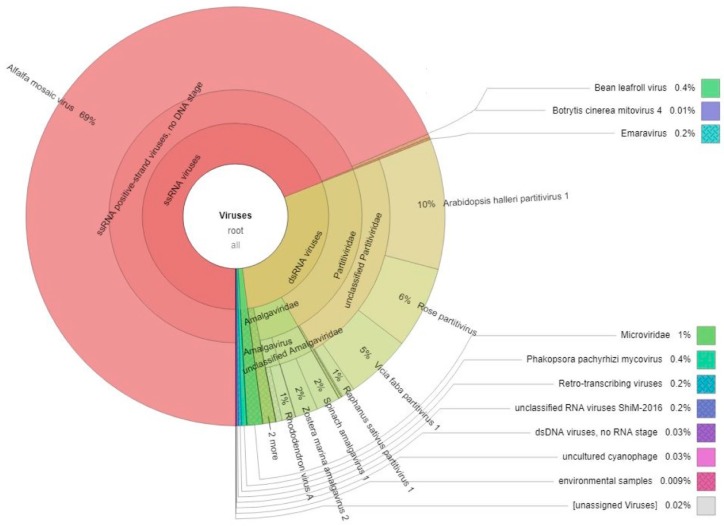
Krona plot representing an overview of all viral sequences identified by Illumina sequencing of immuno-captured alfalfa double-stranded RNA.

Barcoded cDNAs of individual alfalfa dsRNA samples were size-selected to range between 300 and 800 bp (Figure S1a), pooled, fragmented into libraries, and sequenced on Illumina MiSeq (Illumina Inc., San Diego, CA, USA). From the total HTS data, 70.5% of nucleotides passed a high-quality score (>Q30) using FASTQC. The raw reads of pooled tagged cDNAs were de-multiplexed and linked to the original samples. The number of reads obtained per individual sample varied between 74,794 and 97,815 (Table 1). De novo assembly of reads using SPAdes genome assembler yielded 140–378 contigs longer than 200 nucleotides per sample (Table 1). Basic Local Alignment Search Tool (BLASTx) searches against a local database of nonredundant viral proteins identified contigs of putative viral origin with sequence identities of 53–100% to the genome sequences of five different plant viruses: AMV, BLRV, an emara-like virus (genus *Emaravirus*, family *Fimoviridae*, order *Bunyavirales*), and the persistent viruses *Medicago sativa* alphapartitivirus 1 (MsAPV1) and *Medicago sativa* amalgavirus 1 (MsAV1) (Table 1). Taxonomic sequence profiles based on Kaiju [46] were done on all the reads pooled together using the bacteria, archaea, and virus National Center for Biotechnology Information (NCBI) RefSeq databases and were visualized by Krona graph [47]. Mapping, BLAST, and Kaiju approaches provided complementary data support with 100% convergence. The majority of viral sequences were derived from AMV, a single-strand (ss), positive-sense RNA virus that constituted 99% of the total ssRNA viruses (Figure 1). RNA viruses, with either positive or negative-sense genomes, represented 69% of the total viral reads (5% of total FASTQ reads). Persistent dsRNA viruses were represented by 29% of the total viral reads and 2% of the total FASTQ reads (Figure 1). Based on Krona analysis, only 0.4% of ssRNA viral reads were attributed to BLRV (0.02% of total FASTQ reads, Figure 1) and emaravirus-like sequences constituted 0.2% of ssRNA virus reads (0.01% of total FASTQ reads, Figure 1). 

**Figure 2:**
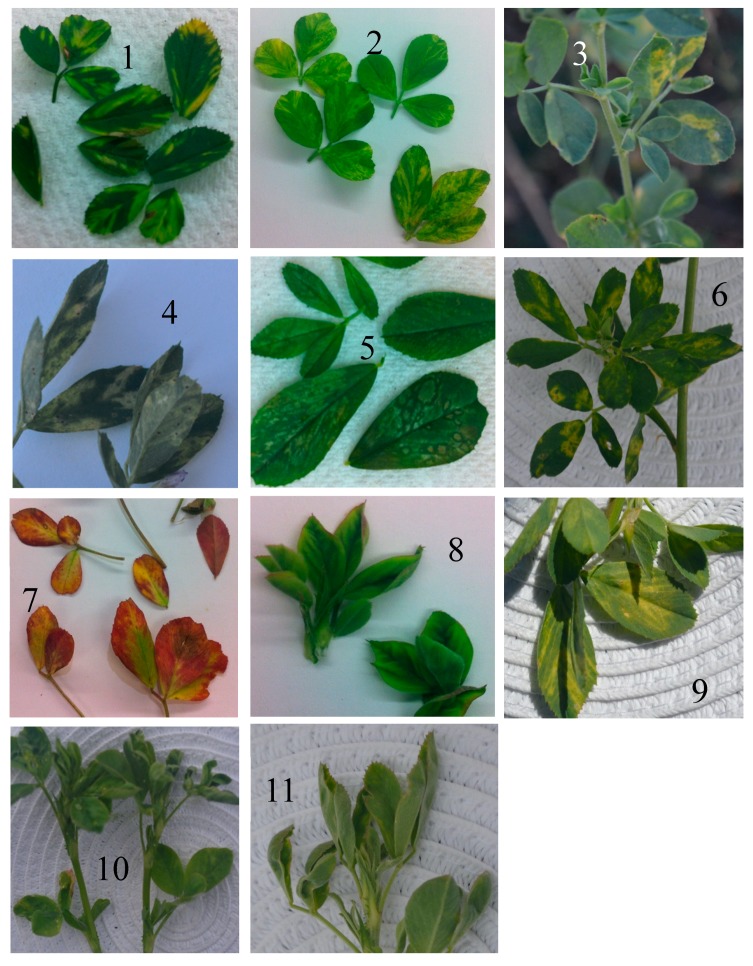
Alfalfa leaves with virus-like symptoms used for RNA virome analysis (samples 1–10) and DNA virus analysis (sample 11): See Table 2 for origin of samples, symptom description, and presence or absence of viruses. Sample 11 is infected by chickpea chlorosis Australia virus and alfalfa mosaic virus and shows leaf curling and minor enations.

BLASTx searches of reads belonging to samples 1 and 8 (Figure 2) as well as Krona plotting revealed two assembled contigs of ~420 nucleotides (nt) with similarity to dsDNA viruses from the *Caulimoviridae* family, sharing 43% and 34% nt sequence identity with the reverse transcriptase gene of soybean chlorotic mottle virus and cauliflower mosaic virus, respectively (Figure 1). However, rolling circle amplification (RCA) followed by restriction enzyme digestions did not confirm the presence of caulimovirus-like DNA in these samples. The abundance of viral reads per sample varied from 1.5% to 25.2% (Table 1). The remaining reads (75%) mainly mapped to chloroplast sequences of *Medicago sativa* (GenBank KU321683).

Restriction enzyme digests of RCA products derived from thirty-four selected symptomatic alfalfa samples (leaf curling and minor leaf enation) using *Eco*RI yielded a ~3-kb band, indicative of a full-length DNA virus genome, only from sample 11 (Figure S1b,c) which displayed symptoms of leaf rolling and mild yellowing (Figure 2). *Hind*III, *Bam*HI, and *Nco*I did not cleave any of the RCA products, and digestion with *Aci*I resulted in DNA fragments smaller than 800 bp in three samples, inconsistent with expected genome sizes of known *Medicago*-infecting geminiviruses. The viral nt sequence identified in sample 11 of 2574 bp was 97% identical to the genome of the known mastrevirus (family *Geminiviridae*) chickpea chlorosis Australia virus (CpCAV) (GenBank KC172686). 

### 2.2. Genome Assembly and Sequence Comparisons of AMV and BLRV Isolates

In all tested alfalfa samples, BLAST analysis of contigs ranging from 1706 nt to 3583 nt showed 95–100% nt sequence identity to RNAs 1, 2, and 3 of AMV isolates on GenBank (Table 1 and Table 2, and Table S2). The presence of AMV RNA in all 10 samples was confirmed by RT-PCR of the coat protein (CP) gene (Table 2 and Figure S2a). The CP amplicon sequences of all samples were 100% identical to the reference sequence (GenBank KC881010; Manfredi isolate from Argentina). The complete sequences of RNA1, RNA2, and RNA3 of AMV were assembled from sample 1 (Figure 2; collected in the State of Victoria) reads (Table 2 and Table S2). This genome represents the first complete sequence of an Australian AMV isolate (AMV-AU) from alfalfa. A total of 18,616 reads were mapped to AMV-Manfredi RNAs 1, 2 and 3 (KC881008-KC881010) as a reference: 12,631 reads correspond to RNA1, 2292 correspond to RNA2, and 3693 correspond to RNA3, with average fold-coverage of 2876, 784, and 517, respectively (Table S1). The sequences of AMV-AU genome segments comprised 3643 nt for RNA1, 2592 nt for RNA2, and 2038 nt for RNA 3 (Genbank MK648424 to MK648426). AMV-AU nt sequence identity with other AMV isolates was 96.6–99.2% for RNA1, 95.2–97.8% for RNA2, and 95.2–99.4% for RNA3 (Table S2). In phylogenetic analyses of RNAs 1, 2, and 3, AMV-AU formed a tight cluster with cognate AMV genome sequences deposited in GenBank (data not shown), similar to an earlier CP sequence phylogenetic analysis of Australian and global AMV isolates [45]. Nucleotide sequence identities between the Victorian AMV isolates from alfalfa and field pea [48] were 99.1%, 94.7%, and 99.3% for RNAs 1, 2 and 3, respectively (Table S2). Sequence identities of both nt and deduced amino acid (aa) sequences for Protein 1 (P1), Protein 2 (P2), Movement Protein (MP), and CP genes ranged from 94.2% to 100% (Table S2). AMV-AU exhibited the highest sequence identity (>99% for nt and aa sequences) in RNAs 1 and 3 with the Manfredi isolate from Argentina. AMV-AU RNA2 sequence identity was highest with isolates from USA and Canada (Table S2).

Due to the low number of reads (146) that mapped to BLRV, the complete sequence of BLRV genome could not be assembled from sample 10, the only sample infected with this virus in this study. Unresolved genome regions included the RNA-dependent RNA polymerase (RdRP), CP and movement protein genes, and the 3’ end of the intergenic region between open reading frames (ORF) 2 and 3 (Table 1 and Table S1). The BLRV sequences obtained covered only 36.7% of the complete BLRV reference genome (Table 1 and Table S1). The presence of BLRV RNA in sample 10 (Figure 2) was however confirmed by RT-PCR using CP-specific primers (Figure S2b) and direct amplicon sequencing, showing 100% sequence identity with known BLRV sequences in BLAST searches (data not shown) 

### 2.3. A putative New Emaravirus Associated with Alfalfa

BLASTx analysis of the assembled contigs from the dsRNA-enriched library reads revealed that some contigs in samples 2, 3, 5, and 9 were most similar to emaravirus (family *Fimoviridae*, order *Bunyavirales*) sequences available in GenBank (Table 1). Emaraviruses are enveloped plant viruses with segmented, linear, negative-sense RNA genomes [49]. These samples were collected in South Australia and Victoria, and all showed ringspot-like symptoms (Figure 2). These contigs ranged in size between 319 and 1069 nt and shared 56–96% aa sequence identity with the RdRP, nucleocapsid (NC), and movement (MP) proteins (RNA1, RNA3, and RNA4, respectively) of several emaraviruses including High Plains wheat mosaic virus (HPWMoV RNA3, GenBank KJ939625) and RNA4 (GenBank KJ939627), wheat mosaic virus (WMoV RNA 1, GenBank KT988860), raspberry leaf blotch virus RNA3 (RLBV, GenBank FR823301), and fig mosaic virus RNA3 (GenBank MG880757). The related emaravirus-like contigs in each sample were assembled de novo, and individual trimmed reads were mapped back to the assembled emaravirus-like consensus fragments and reassembled until all reads were incorporated (Table 1 and Table S3).

**Figure 3:**
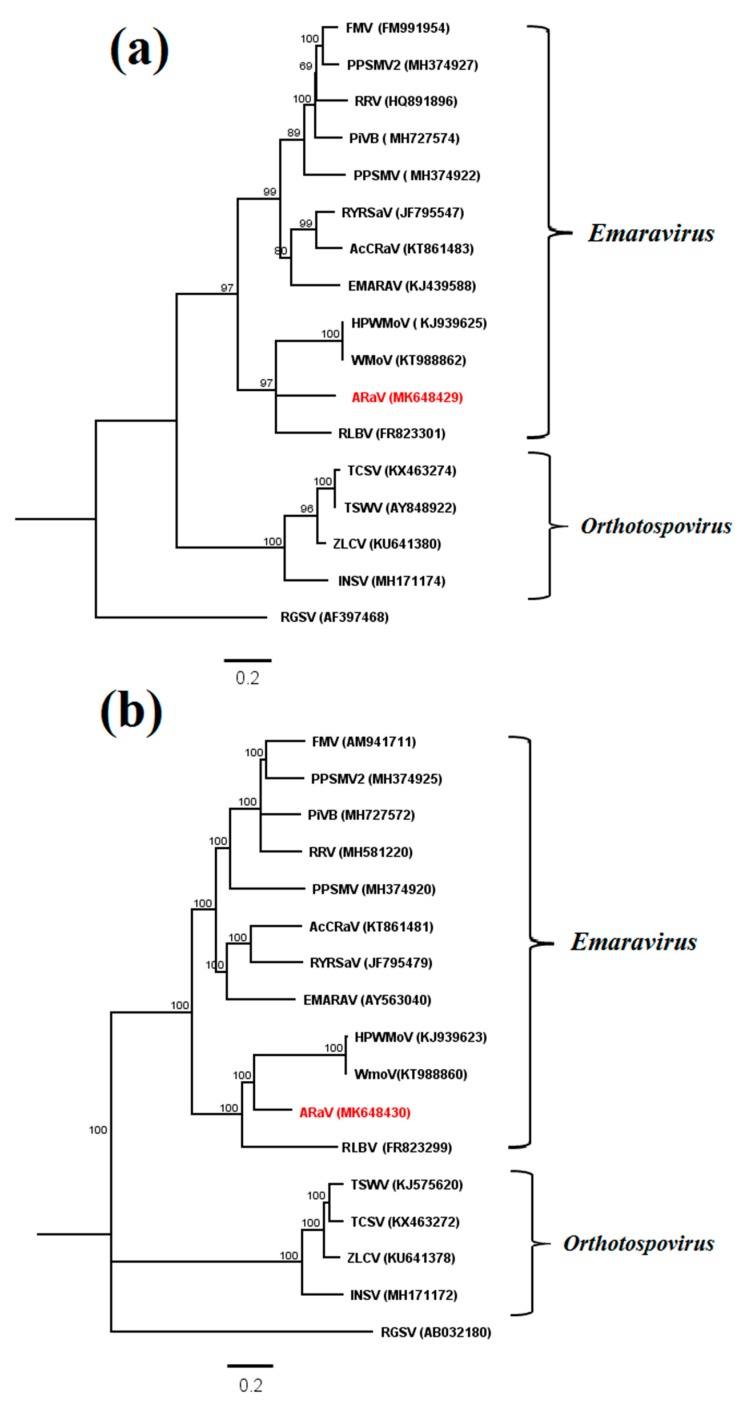
Phylogenetic analyses of alfalfa ringspot-associated virus (ARaV) in relation to emaraviruses and selected orthotospoviruses in the order *Bunyavirales*: Neighbor-joining phylograms of (**a**) complete nucleoprotein amino acid sequences and (**b**) partial L protein amino acid sequences. Virus names and GenBank accession numbers are listed in Table S8. Bootstrap values are shown as a percentages of 1000 replications. The scale bar indicates the number of substitutions per site. Rice grassy stunt virus (RGSV; genus *Tenuivirus*) was used as an outgroup to root the trees.

**Figure 4:**
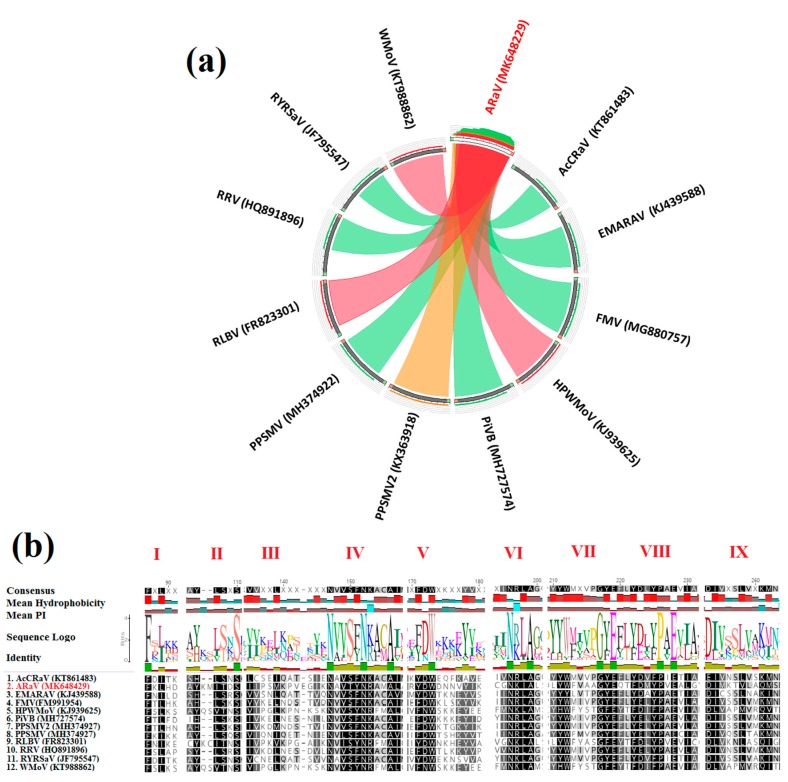
Sequence identity between alfalfa ringspot-associated virus (ARaV, red) and known emaraviruses: (**a**) Visual representation of amino acid sequence relationships of ARaV nucleoprotein with cognate sequences of emaraviruses by Circoletto diagram [50]. The ribbons represent local BLAST alignments, their width represents the alignment length, and colors indicate the percentage of maximum bitscores: green (25–50%), orange (50–75%), and red (75–100%). (**b**) Amino acid sequence alignment of conserved nucleoprotein motifs I–IX of ARaV and selected emaraviruses: Virus names and GenBank accession numbers are listed in Table S8.

The emaravirus-like contigs identified in sample 2 were further analyzed. They included three nonoverlapping contigs that mapped to RNA1 and partial sequences of RNA3 and RNA4 of a putative new emaravirus (Table S3) that was tentatively named alfalfa ringspot-associated virus (ARaV). No contigs were found that mapped to emaravirus RNA2. To resolve the apparent sequence gaps in RNA1 of ARaV, bridging primers were designed for RT-PCR using the total RNA extract of sample 2 and the amplicons were cloned and sequenced (Table S4). The assembled partial RNA1 sequence of ARaV was 2787 nt. The sequences of the partial RdRP ORF (2723 nt) on RNA1 and the complete NC protein ORF on RNA3 (903 nt and 300 aa) were retrieved and phylogenetically analyzed. In neighbor-joining (NJ) phylograms, ARaV clustered with known emaraviruses, most closely with WMoV, HPWMoV, and RLBV, and separate from the orthotospovirus clade (Figure 3a,b). Similar tree topologies were obtained when maximum likelihood phylograms were generated for the same dataset (data not shown). The close sequence relationship between ARaV and emaraviruses was further visualized in a Circoletto diagram based on BLASTp alignment bit-scores [50]. ARaV N protein aa sequence had the highest bit-score similarity of 90–96% with N protein of the emaraviruses RLBV, HPWMoV, and WMoV (Figure 4a). Multiple sequence comparison by log-expectation (MUSCLE) alignment of emaravirus N protein aa sequences showed that ARaV also contains nine motifs that are conserved across the N protein sequences of known emaraviruses (Figure 4b). Furthermore, the predicted 5′ untranslated region (UTR) secondary structures of ARaV, HPWMoV, and WMoV RNA1 (GenBank MK648430, KJ939623, and KT988860, respectively) can form similar loop regions (Figure S3) that may be involved in replication. Taken together, these data provide initial molecular evidence of the first emaravirus associated with alfalfa. Sequence Demarcation Tool analysis shows that ARaV NC aa sequence differs from that of all other known emaraviruses by more than 50% (data not shown). Species demarcation in the genus *Emaravirus* is based on aa sequence difference of more than 25% for cognate proteins encoded on the respective viral RNAs [51], suggesting that ARaV should be considered as a new species.

### 2.4. Persistent dsRNA Viruses Associated with Alfalfa

The results of BLASTn and BLASTx searches of de novo assembled HTS contigs indicated the presence of sequences with strong similarities to persistent dsRNA viruses in the families *Amalgaviridae* and *Partitiviridae* in all alfalfa samples (Table 1 and Table S5). Most of these viral reads corresponded to *Medicago sativa* alphapartitivirus 1 (MsAPV1) RdRP (MK292286), and CP (MK292287), and *Medicago sativa* amalgavirus 1 (MsAV1; BK010406), with a genome coverage of 16 to 2657-fold, 16 to 737-fold, and 8 to 836-fold, respectively (Table S5). Presence of MsAV1 in samples 2, 6, and 9 was confirmed by RT-PCR, and MsAPV1-dsRNA1 amplicons were obtained from samples 2, 5, 7, 9, and 10. Virus titers in the other samples were too low for detection by gel-based RT-PCR analysis (Figure S2c–e). The complete genome sequences of MsAV1-AU (3423 bp), MsAPV1-dsRNA1 (1868 bp), and MsAPV1-dsRNA2 (1806 bp) were assembled from sample 9 (Figure S4) and deposited in GenBank with accession numbers MK648427, MK648428, and MN660231.

#### 2.4.1. *Medicago sativa* Alphapartitivirus 1

**Figure 5:**
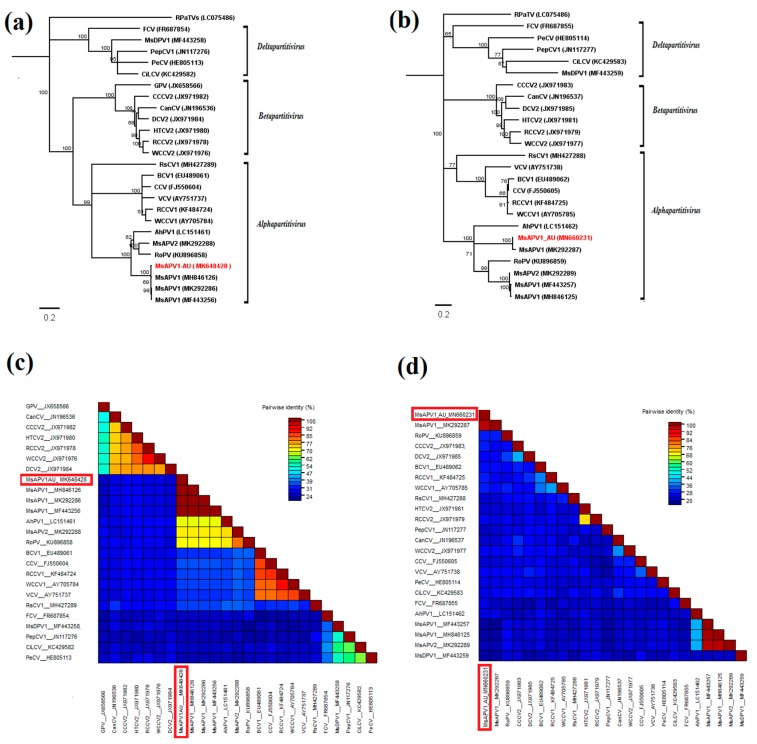
Neighbor-joining (NJ) phylograms of MsAPV1-AU and other members of the family *Partitiviridae* based on (**a**) RNA-dependent RNA polymerase (RdRP; dsRNA1) and (**b**) coat protein (CP; dsRNA2) amino acid sequences: Bootstrap values are shown as percentages of 1000 replications. The scale bar indicates the number of substitutions per site. Red clover powdery mildew-associated totivirus, RPaTV, was used as the outgroup. Virus names and GenBank accession numbers are listed in Table S7. The topologies from NJ and maximum likelihood (not shown) analyses were identical. Pairwise identities plots of (**c**) RdRP and (**d**) CP of partitiviruses aligned by MUSCLE and displayed by Sequence Demarcation Tool software: Virus names and GenBank accession numbers are listed in Table S7. Colored heat map of pairwise identities corresponds to the colors on the scale.

Most alphapartitivirus-like contigs shared 100% sequence identity with MsAPV1-dsRNA1. BLASTx searches also indicated the presence of MsAPV1-dsRNA2. The presence of dsRNA2 was confirmed in all samples by RT-PCR, using MsAPV1 CP-specific primers. Amplicon yields were generally low, suggesting low viral titres, except in sample 10 (Figure S2d). MsAPV1 dsRNA1 contained a single ORF from nt 101 to nt 1861 encoding the putative RdRP of 586 aa with an estimated molecular mass of 68.7 kDa and short 5’ and 3’ UTRs (Figure S4a). MsAPV1 dsRNA2 contained a single ORF from nt 96 to nt 1595 encoding the putative CP of 499 aa with an estimated molecular mass of 55.4 kDa and a short 5’ UTR and longer 3’ UTR (Figure S4b). As is the case for all known plant partitiviruses, a stem loop-like structure is predicted for the 5’ UTR of dsRNA1 of the Australian MsAPV1 (MsAPV1-AU) (Figure S5a), which has been proposed to play a role in dsRNA replication and particle assembly [18]. NJ phylograms show that MsAPV1-AU dsRNA1 sequence is closely related to plant alphapartitiviruses, most closely related to MsAPV1 sequences from Argentina, USA, and China (Figure 5a). The phylogenies based on CP and RdRP genome segments of MsAPV1-AU were congruent (Figure 5a,b). Overall, MsAPV1-AU RdRP had 40–100% aa sequence identity with other alphapartitiviruses and 24–40% with other family members (deltapartitiviruses and betapartitiviruses) (Figure 5c). MsAPV1-AU RdRP aa sequence was 100% identical to RdRP sequences of all other available MsAPV1 isolates but only 70% identical to that of the distinct alphapartitivirus MsAPV2 (Figure 5c). MsAPV1-AU CP showed 42–100% aa sequence identity with other alphapartitiviruses and 20–40% with deltapartitiviruses and betapartitiviruses (Figure 5d). MsAPV1-AU CP aa sequence was 98% identical to that of MsAPV1-Manfredi isolate but only 21% identical to that of MsAPV2 (Figure 5d).

#### 2.4.2. *Medicago sativa* Amalgavirus 1

**Figure 6:**
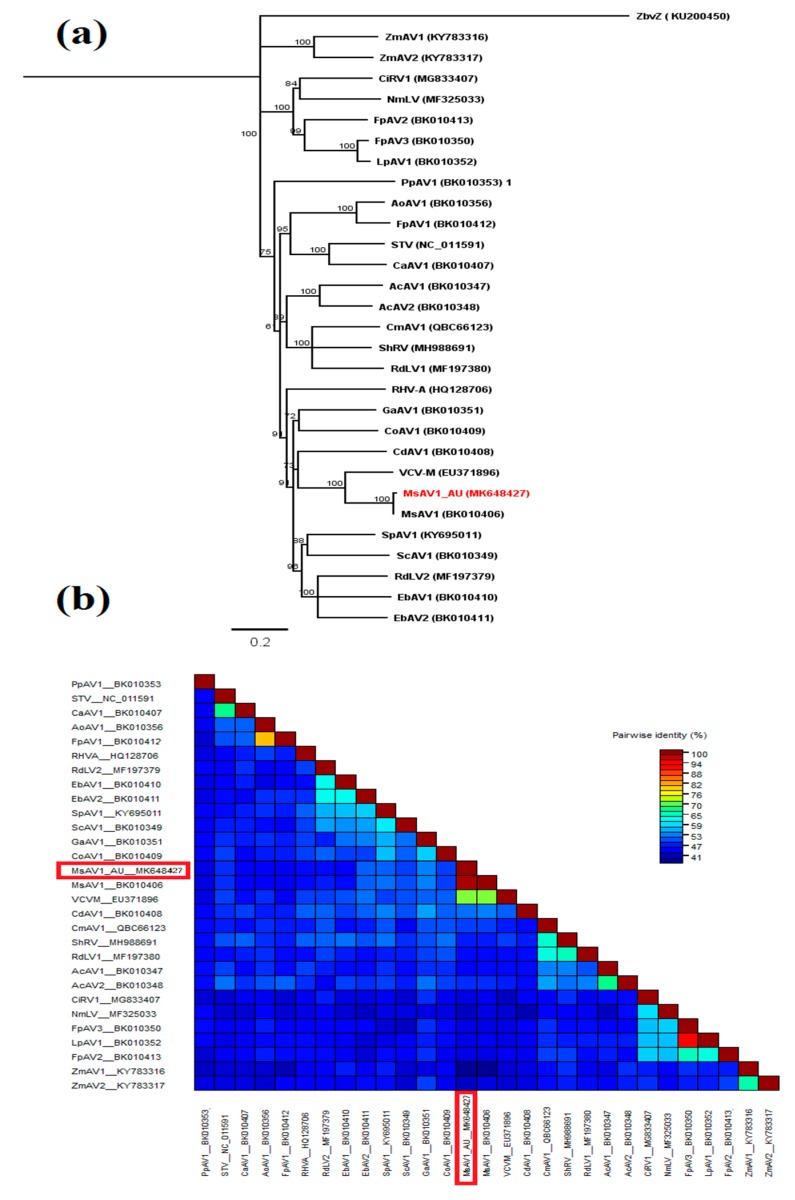
Phylogenetic and sequence identity analysis of *Medicago sativa* amalgavirus 1 (MsAV1-AU): (**a**) Phylogenetic relationships of MsAV1-AU and other members of the family *Amalgaviridae* based on fusion protein (FP) amino acid sequences. Bootstrap values of neighbor-joining (NJ) phylogram are shown as percentages of 1000 replications. The scale bar indicates the number of substitutions per site. *Zygosaccharomyces bailii* virus Z (ZbvZ) was used as the outgroup. The topologies from NJ and maximum likelihood (not shown) analyses were identical. (**b**) Pairwise identities plot of FPs of amalgaviruses aligned by MUSCLE and displayed by Sequence Demarcation Tool software: Colored heat map of pairwise identities as shown on the scale. Virus names and GenBank accession numbers are listed in Table S7.

The dsRNA genome of MsAV1 from South Australia (MsAV1-AU) contains two partially overlapping ORFs in the positive strand (Figure S4c). ORF1 starts at nt 130 and ends at nt 1314, encoding a putative CP of 394 aa with a calculated molecular mass of 44.0 kDa. As is the case for all known plant amalgaviruses, the predicted secondary structure of the CP features a typical N-terminal α-helix between aa positions 24 and 153 (Figure S6). The predicted read-through ORF1+2 in MsAV1-AU starts at 130 nt and ends at 3307 nt, encoding a putative fusion protein (FP) of 1058 aa with an estimated molecular mass of 111 kDa (Figure S4c). The 5’ and 3’ UTRs are 129 nt and 116 nt, respectively; AU-rich (59.7% and 59.5%, respectively), and capable of forming complex secondary stem loop-like structures (Figure S5b,c), which may assist RdRP recognition during viral replication [52]. The FP aa sequence placed MsAV1-AU in a large phylogenetic clade with other known plant amalgaviruses, most closely related to MsAV1-Maverick (BK010406) (Figure 6a). FP of MsAV1-AU shared 54.5–99% aa sequence identity with FPs of other amalgaviruses and had the highest (99%) sequence identity with FP of MsAV1-Maverick, demonstrating a close genetic relationship between isolates of this amalgavirus (Figure 6b).

### 2.5. Chickpea Chlorosis Australia Virus

**Figure 7:**
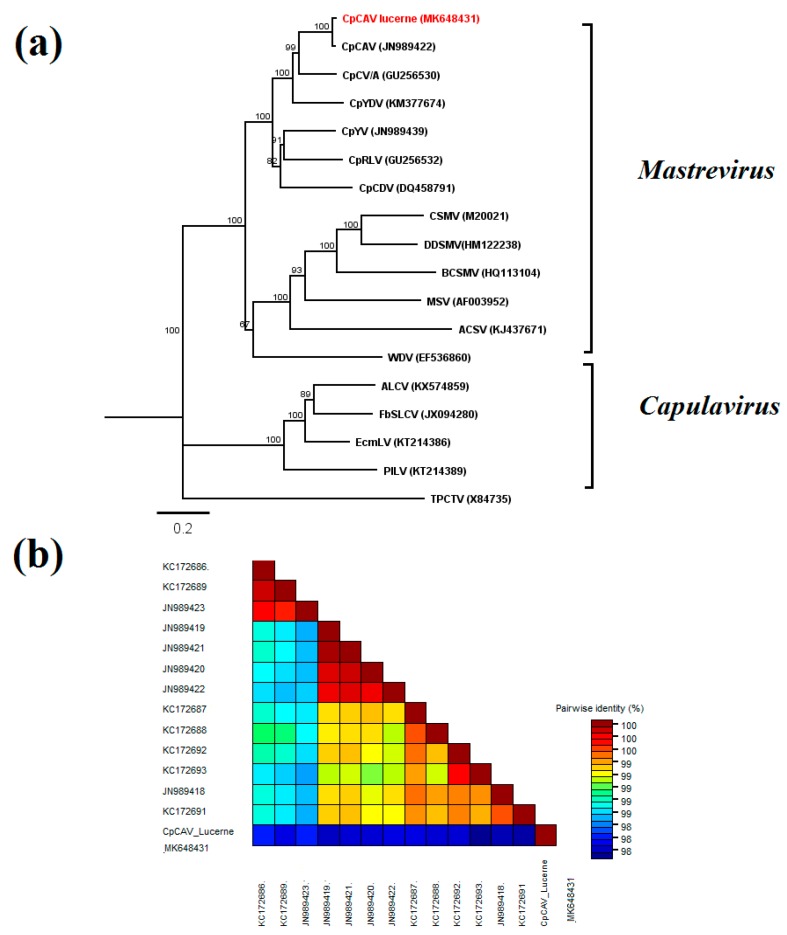
Phylogenetic relationships and pairwise identities of chickpea chlorosis Australia virus (CpCAV) and selected geminiviruses: (**a**) Phylogenetic relationships of CpCAV from alfalfa and selected mastreviruses and capulaviruses based on complete genome sequences. Bootstrap values of neighbor-joining (NJ) phylogram are shown as percentages of 1000 replications. The scale bar indicates the number of substitutions per site. Tomato pseudo-curly top virus (TpCTV) was selected as the outgroup. The topologies from NJ and maximum likelihood (not shown) were identical. (**b**) Genome-wide pairwise identities among complete CpCAV genomes determined by Sequence Demarcation Tool: The colored heat map of 98–100% pairwise identities indicates high similarity of all known CpCAV sequences. Virus names and GenBank accession numbers are listed in Tables S9 and S10.

**Figure 8:**
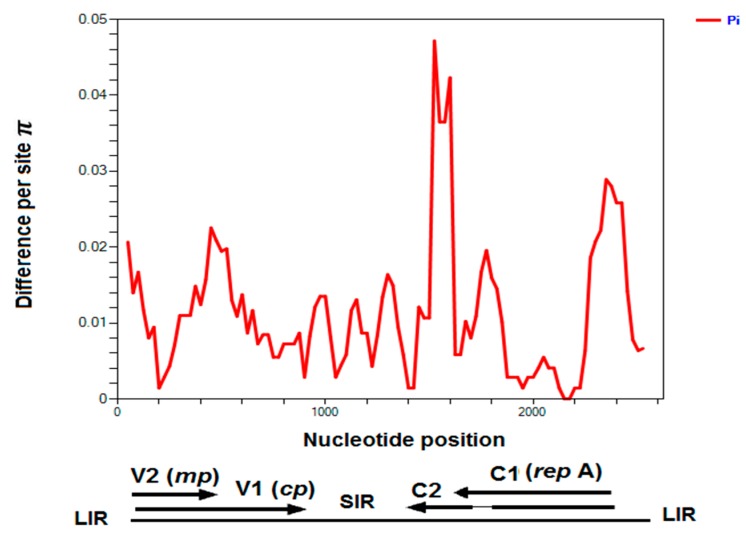
Extent and distribution of genetic variation along CpCAV complete genome sequences (Table S10) estimated by nucleotide diversity Pi: A 100-nt sliding window was used with a 25-nt step size. Virion-sense genes V1 (cp-coat protein) and V2 (mp-movement protein) and complementary-sense genes C1 and C2 are indicated, along with the long and short intergenic regions (LIR and SIR) in this linearized depiction of the circular DNA.

Chickpea chlorosis Australia virus (CpCAV) was identified in alfalfa sample 11 from South Australia, which was also infected by AMV and showed leaf rolling and yellowing symptoms (Figure 2). The complete ssDNA genome of 2575 nt was sequenced. This is the first report of alfalfa as a host for this virus. Phylogenetic analysis of this CpCAV alfalfa isolate compared to representative mastreviruses and capulaviruses revealed two major distinct, well-supported clades, one that included all mastreviruses and another that contains the capulaviruses including ALCV Manfredi that is associated with ADD in Argentina (Figure 7a). The CpCAV alfalfa isolate grouped with the mastreviruses and appears to be most closely related to a CpCAV isolate collected in New South Wales in 2002 from chickpea (*Cicer arietinum*) (Figure 7a). The known CpCAV isolates available in GenBank share >97.6% genome-wide pairwise identity, and the alfalfa isolate was no different (Figure 7b). Nucleotide sequence diversity analysis among 14 CpCAV isolates identified 104 polymorphic sites. The variation rates were unevenly distributed across the genome with the main peak adjacent to the 3’ end of the overlapped C2 ORF (nt 1600) (Figure 8).

## 3. Discussion

We identified several known and one novel RNA virus through HTS of 10 alfalfa samples in Australia that showed a range of virus-like symptoms including yellow mosaic, ring spots, and leaf curling. We also identified and determined the complete genome of the ssDNA virus CpCAV from another alfalfa sample with leaf curl/roll-like symptoms following rolling circle amplification. The identified RNA viruses included the endemic AMV and BLRV [3,5,45], a new putative emaravirus, and two persistent dsRNA viruses in the *Amalgaviridae* and *Partitiviridae* families, previously reported in alfalfa from other countries [16,17,18,52,53]. The majority of samples and viruses identified by HTS were confirmed by RT-PCR (Table 2). This is true for all samples in the case of AMV and BLRV and all but one sample for ARaV. However, the low titre cryptic dsRNA viruses MsAV1 and MsAPV1 were sometimes not detectable by gel-based RT-PCR due to lower sensitivity than HTS. Based on their biology, cryptic viruses can be expected to occur in all the samples and are known to show little sequence variation; we took great care to avoid any cross-contamination. This is the first metagenomics study to assemble genomes of RNA viruses from infected alfalfa in Australia. In this study, we did not detect CMV, lucerne transient streak virus, and a number of luteo- and nepoviruses that had been identified on occasion in Australian lucerne crops in earlier studies [3,4,5,6].

In this study of alfalfa viromes, we sequenced cDNA libraries from immune-captured dsRNA, followed by a bioinformatics pipeline to identify and assemble viral sequences. Viral nucleic acid enrichment in metagenomics has accelerated detection of novel viruses especially in large-scale surveys [20,34,35,36]. Application of monoclonal antibodies as an immune-capture-based dsRNA enrichment approach has provided a rapid alternative to the classical purification of dsRNA using CF11 cellulose [35]. 

AMV was identified in all ten alfalfa samples confirming that this virus was wide-spread in plants grown for seed and hay [45]. In the current study, we assembled the complete genome of AMV from one of those samples, including all 3 genomic RNAs, and this represents the first complete AMV genome sequence from alfalfa and the second complete genome of an Australian AMV isolate. In our previous phylogenetic study, based on the CP encoding gene of AMV-AU from alfalfa, AMV formed a monophyletic group of closely related viruses from various plant species and geographic locations [45].

A small number of diagnostic BLRV reads were found in only one sample. A recent limited survey using CP-specific primers in RT-PCR detected BLRV in 70% of symptomatic samples [45], but this virus was not strongly represented in the samples selected for HTS. Whether enrichment of dsRNA by monoclonal antibody 4G2 has completely captured the BRLV genome remains a point of speculation and requires further optimization and analysis of available HTS data.

Sequence reads that most closely matched emaraviruses were identified in four samples, two from seed paddocks in South Australia and two from hay paddocks in Victoria. Based on current scientific literature and available GenBank data, there are no emaraviruses known to infect alfalfa. Based on all four samples showing ringspot symptoms on the leaves, the associated virus was tentatively named alfalfa ringspot-associated virus (ARaV). Emaraviruses are enveloped, segmented negative-sense RNA plant viruses in the family *Fimoviridae*, order *Bunyavirales* that are transmitted by eriophyid mites [51,53]. Recent reports have indicated an increasing number of emaraviruses with up to eight distinct genome segments [49,54,55,56,57,58]. Emaravirus genome segments have been reported to possess complementary 13-nt consensus sequences at their 5’ and 3’ termini [57,58]. However, searches of the HTS raw data of sample 2 in which most ARaV reads were identified, by mapping and iterative genome assembly, did not find this motif and, therefore, putative additional genome segments, likely due to insufficient sequence coverage (see Table 1). ARaV appears to have at least four segments based on sequence similarities of reads with RNAs 1, 3, and 4 of the most closely related emaraviruses WMoV and RLBV. Future research will attempt to isolate this virus for biological studies on virus symptoms, transmission, and prevalence and to determine the number and complete sequences of all its genome segments. The recently reported PCR primers targeting the conserved 13-nt sequence at the termini of emaravirus genome segments may assist amplification of the complete set of ARaV segments [58].

The sequence coverage for the negative-sense RNA virus ARaV was considerably less than for the positive-sense RNA genomes of AMV and BRLV and the dsRNA genomes of MsAV1 and MsAPV1. The percentage of reads that mapped to a consensus sequence of ARaV was also lower than for the other RNA viruses detected in this study. This concurs with the findings by Weber and collaborators [59] that sufficient amounts of dsRNA are generated from viruses with +ssRNA and dsRNA genomes to allow efficient HTS detection, but this may not be the case for −ssRNA viruses. This was also observed by Blouin and collaborators [35] who detected only +ssRNA viruses by HTS following dsRNA enrichment. Our study supports that −ssRNA viruses, such as emaraviruses, can be detected by HTS of enriched dsRNA but with a significantly reduced efficiency. More recently, HTS of dsRNA derived from a transgenic line of common bean resistant to bean golden mosaic virus but with virus-like symptoms had a lower sequencing coverage for negative-sense RNA viruses, such as a putative cytorhabdovirus, when compared to samples with positive-sense RNA viruses, such as comovirus and carlavirus [60].

Although HTS data suggested a caulimo-like virus may be present in some samples, this was not confirmed by RCA and restriction enzyme digestion. The detected caulimovirus-like sequences may instead be derived from endogenous integrated elements [61]. However, if a caulimo-like virus was present, its lack of detection via dsRNA HTS is not surprising because plant DNA viruses do not generate long dsRNA as part of their life cycle [36]. However, detection of DNA viruses by dsRNA sequencing is possible if total dsRNA is not treated with RNase and DNase [62]. No other DNA virus sequences were detected by dsRNA HTS. However, RCA from total DNA extracts of selected alfalfa samples identified the mastrevirus CpCAV in alfalfa—a new report as an alternative host for this virus. The alfalfa and chickpea isolates of CpCAV have a close evolutionary relationship and high sequence identity. Members of the genus *Mastrevirus* (family *Geminiviridae*) have ssDNA genomes, are transmissible by leafhoppers, and can infect both mono- and eudicotyledonous plants [63,64]. In addition to alfalfa and chickpea, French bean has been shown to be a host for CpCAV [64].

Viruses that are taxonomically classified in the families *Partitiviridae*, *Amalgaviridae*, *Totiviridae*, *Endornaviridae*, and *Chrysoviridae* are known as persistent cytoplasmic plant viruses. Persistent viruses have been shown to infect plants or plant-interacting fungi [65,66,67]. These viruses are only transmissible vertically by seed or gametes; they can infect their hosts for many generations; and unlike acute plant viruses that cause disease symptoms in their host, they do not usually cause disease [65]. The presence of MsAV1 and MsAPV1 in all 10 sequenced alfalfa samples in this study indicates that these persistent viruses are widespread and may have been associated with Australian alfalfa for many years. MsAV1, MsAPV1, and related persistent viruses show low genetic diversity over time. This was highlighted in a recent study indicating that the genome of *Zea mays* chrysovirus had only changed by 3% in 1000 years [68]. Although population studies on persistent viruses are rare, there is some evidence that the divergence of persistent viruses correlates with the divergence of their plant hosts [69].

Previous studies have identified and characterized dsRNA viruses from alfalfa that are associated with persistent, non-symptomatic infections including MsAPV1, MsAPV2, and *Medicago sativa* deltapartitivirus 1 [17,18]. The MsAPV1 RdRP sequences (dsRNA1) detected in this study were more than 98% identical to those of MsAPV1 isolates that were previously reported [16,17,18]. This close relationship between alphapartitiviruses from alfalfa cultivars grown in different continents was also evident when comparing CP sequences representing dsRNA2 [18].

Analysis of a transcriptome dataset also identified persistent dsRNA viruses in other plant species, including *Cucumis melo* cryptic virus, *Cucumis melo* amalgavirus 1 from melon [52], a novel alphapartitivirus *Raphanus sativus* cryptic virus from radish [70], and *Salvia hispanica* amalgavirus 1 from chia [71]. Most of the amalgaviruses that have been reported recently were identified in transcriptome datasets [52,72]. The RdRPs of amalgaviruses are thought to be expressed as ORF1+2 fusion proteins by the +1 programmed ribosomal frameshifting mechanism through a conserved motif (UUU_CGN) that has been found in aa sequences of most amalgaviruses including MsAV1 from Australia. However, some amalgaviruses, such as *Capsicum annuum* amalgavirus and southern tomato virus, do not contain this conserved motif [52,72].

An HTS study of small RNAs from alfalfa in China recently detected AMV, ADV, and ALCV [73] that had previously been identified as components of alfalfa dwarf disease in Argentina [7,8,12]. Recently, a suite of 23 viruses were identified in 655 publicly available alfalfa transcriptome datasets [74]. These included AMV, BLRV, MsAPV1, and MsAV1, which we detected in our study and several other known and some emerging viruses not previously reported in alfalfa [74]. 

Our study is the first dsRNA-based HTS virome analysis reported for alfalfa in Australia. In addition to the endemic AMV and BLRV, we have identified −ssRNA genome segments associated with a new emaravirus and dsRNA genomes of an alphapartitivirus and an amalgavirus. RCA and restriction enzyme analysis also revealed alfalfa as an alternative host of chickpea chlorosis Australia virus.

## 4. Materials and Methods

### 4.1. Plant Material for Analysis

Alfalfa leaf samples with diverse virus-like symptoms including leaf curling, yellow mosaic, ring spots, leaf enation, and reddening were selected from a 2015–2017 collection of Australian samples from seed crops near Keith, South Australia and from fodder crops from Hamilton and Edenhope, Victoria [45]. All samples were stored at −80 °C. 

### 4.2. dsRNA Capture

DsRNA was purified from freeze-dried alfalfa leaf samples as described by Blouin and collaborators [35] with minor modifications: Freeze-dried leaf tissue, 100 mg per sample, was ground to a fine powder using a Tissue Lyser II (Qiagen, Chadstone, VIC, Australia). Then, 900 μL of Tris-Buffered Saline with Tween^®^ 20 (TBST) was added, vortexed, and centrifuged at 10,000 rpm to remove plant debris. Pierce protein L magnetic beads (Thermo Fisher Scientific, Scoresby, VIC, Australia) (10 μg/ul) were washed two times with 150 μL and 1000 μL, respectively, of TBST as per the manufacturer’s protocol and were then coated with 500 μL of dsRNA-specific mAb 4G2 [75] and incubated on a rotary shaker at room temperature for 1 h. The beads were collected with a magnetic stand, the supernatant was discarded, and the beads were washed with 500 μL of TBST before incubation with 500 μL of clarified plant extract on a rotary shaker for 1 h. After collection of beads, the supernatant was removed, and the beads were washed twice with TBST and then resuspended in 30 μL of ultrapure nuclease-free water.

### 4.3. High Throughput Sequencing of dsRNA

For reverse transcription (RT), 6 μL of the resuspended dsRNA-bound beads was mixed with 3 μL of 20 μM random N6 primer (5′-CCTTCGGATCCTCCNNNNNN-3′) and 9 μL of nuclease-free ultrapure water and heated at 95 °C for 2 min followed by immediate cooling on ice for 1 min. After denaturation, 300 units (1.5 μL) of SuperScript III reverse transcriptase (Thermo Fisher Scientific), 1.5 μL of 10 mM deoxyribonucleotide triphosphate, 6 μL of 5 × buffer, and 3 μL of 100 mM dithiothreitol earlier mixed in a separate PCR tube were added to the RT-mix. The mix was kept on ice for 15 min and then incubated at 50 °C for 1 h. This was followed by treatment with 0.375 μL ribonuclease A (20 mL/mg; Sigma-Aldrich, Sydney, NSW, Australia) at room temperature for 15 min and enzyme inactivation at 85 °C for 2 min. The cDNA was column-purified using the PCR Clean-Up System (Promega, Alexandria, NSW, Australia) and eluted in 30 μL of ultrapure nuclease-free water.

cDNA was amplified by PCR and individual barcodes (Table S6) incorporated using CloneAmp™ HiFi PCR Premix Polymerase (Clontech—Takara Bio, Mountain View, CA, USA) as per the manufacturer’s protocol with 4 μL of cDNA, 10 μL of 2 × buffer HiFi PCR Premix, 1 μL of 10 μM single barcode, and 5 μL of ultrapure nuclease-free water for a reaction volume of 20 μL for rapid amplification two-step reactions. The first step comprised 98 °C for 2 min, 65 °C for 1 min, and 72 °C for 1 min, and the second step consisted of 40 cycles of 98 °C for 5 s, 55–58.3°C for 5 s (optimized by gradient PCR for individual barcodes), and 72 °C for 5 s followed by a final extension at 72 °C for 5 min. Samples were loaded on a 1% agarose gel, and the smear between 300 bp and 800 bp was excised and purified by PCR Clean-Up System. Each barcoded-sample was quantified using Qubit fluorimetric quantification (Thermo Fisher Scientific), and the integrity of samples was determined using the Agilent 2100 Bioanalyzer (Agilent Technologies, Mulgrave, VIC, Australia). Library preparation of the combined samples (60 ng each) using the NEBNext^®^ Ultra^™^ DNA Library Prep Kit for Illumina^®^ (New England Biolabs—NEB, Ipswich, MA, USA) and HTS were done by Genewiz, Inc. (Suzhou, China) on an Illumina MiSeq in a 2 × 250 bp paired-end format.

### 4.4. Identification of DNA Viruses

Viral DNA genomes were amplified by RCA from total DNA extracts [76] using phi29 DNA polymerase following the manufacturer’s protocol (Illustra TempliPhi, GE Healthcare, Parramatta, NSW, Australia). The RCA genomic concatemers were visualized on 1% agarose/TBE gel, and aliquots were linearized with *Eco*RI, gel-purified and ligated using T4 DNA ligase (NEB) into pUC19 that had been linearized by *Eco*RI digestion and dephosphorylated using Antarctic Phosphatase (NEB). Three recombinant clones per amplicon were sequenced in both directions at the Australian Genome Research Facility (AGRF, Brisbane, Australia) using M13F and M13R and internal primers. Trimmed sequence reads were assembled into contigs and a consensus sequence generated for each cloned DNA using Geneious R 9.1 (Biomatters Ltd., Auckland, New Zealand). Tomato yellow leaf curl virus (TYLCV, genus *Begomovirus*, family *Geminiviridae*) was used as a positive control. Individual consensus nucleotide sequences were searched against GenBank databases via BLASTn and BLASTx and mapped to the closest reference sequence using Geneious.

### 4.5. Bioinformatics Analyses

HTS reads were analyzed in the Galaxy web-based platform (https://usegalaxy.org/). Trimmomatic was used with default parameter settings to remove low-quality reads from the Illumina sequence data, and read quality was assessed by FASTQC (http://www.bioinformatics.babraham.ac.uk/projects/fastqc/). Using barcode splitter, (https://toolshedg2.bx.psu.edu/view/devteam/fastx_barcode_splitter), individual sequence reads were de-multiplexed. Barcodes listed in Table S6 were trimmed from reads, and the forward and reverse sequences were concatenated. Contigs were assembled de novo using SPAdes genome assembler online with default parameters [77]. The resulting contigs as FASTA files were compared to the viral RefSeq database using BLASTx in Geneious. All contigs that matched viral sequences were then used in a BLASTx search against the GenBank nonredundant database and BLASTn to confirm viral contigs and to exclude any false positives (e.g., cellular RNA-dependent RNA polymerases). Taxonomic profiling was performed on pooled FastQ reads that were compressed with GZIP (fastq.gz file) by Kaiju (http://kaiju.binf.ku.dk/) [46] using the NCBI RefSeq database (last updated June 2019), and results were visualized by Krona tool [47].

### 4.6. Verification of HTS-Based Virus Detection by RT-PCR and Sanger Sequencing

The identity of virus-like sequences detected by HTS was verified by RT-PCR using primers designed from the viral contigs and targeting RdRP and CP or NC genes of the viral genomes (Table S4). RT-PCR was done with total RNA extracted from 100 mg of individual leaf samples using a RNeasy Plant Mini Kit (Qiagen), 10 μM of each primer and Superscript III One-Step RT-PCR kit with Platinum^®^ *Taq* DNA polymerase (Thermo Fisher Scientific) following the manufacturer’s protocol. RT-PCR conditions were 50 °C for 30 min, 94 °C for 2 min, 35 cycles of 94 °C for 15 s, 55 °C for 30 s, and 68 °C for 1 min, followed by a final extension at 68 °C for 5 min. Amplicons were analyzed on 1% agarose/TBE gel, DNA bands stained using Redsafe™ (iNtRON Biotechnology, South Korea) and visualized under UV light using a gel documentation system (BioRad, Gladesville, NSW, Australia). DNA bands were excised, gel-purified using Wizard^®^ DNA clean up system (Promega), and quantified by Nanodrop^®^ spectrophotometer. Amplicons were either sequenced directly or first ligated into pGEM-T Easy (Promega) and sequenced at the AGRF using M13 forward and reverse primers.

### 4.7. Phylogenetic and Sequence Diversity Analysis

Sequences of type isolates and representatives of phylogroups related to the viruses that were identified during HTS analysis in this study were retrieved from GenBank for phylogenetic analyses. De novo assembled contigs were subjected to bulk BLASTn and BLASTx searches. Reference assemblies of complete or partial viral genomes from reads of the individual ten barcoded libraries were done using Geneious with default parameters, and the reads were mapped against the most similar viral genomes identified in BLASTn and BLASTx. Multiple sequence alignments based on the complete viral genomes, NC, CP, and RdRP nucleotide and amino acid consensus sequences of the viruses identified in this study and published sequences available on GenBank were generated by MUSCLE, and conserved domains prediction of the CP and RdRP were done using Geneious. Phylogenetic trees were inferred from the aligned sequences using the NJ method implemented in Geneious with 1000 bootstrap replicates, and branches with less than 60% bootstrap support were collapsed. Pairwise identity analyses of the viral genome sequences were implemented by the MUSCLE-based pairwise alignment option using Sequence Demarcation Tool (SDT v1.2) [78]. For CpCAV isolates (Table S10), the average number of nucleotide difference per site (π) was calculated with a sliding window of 100 nts and a 25 nt step size, using DNA sequence polymorphism (DnaSP v5) [79].

RNA secondary structure was predicted with RNA structure prediction tool [80] available online at http://rna.urmc.rochester.edu/RNAstructureWeb/. The protein structure was predicted with Emboss online software (http://www.bioinformatics.nl/cgi-bin/emboss/garnier), which is based on the Garnier Osguthorpe–Robson method [81]. The aa sequence identities of emaravirus NC proteins were expressed as Circoletto diagrams (http://bat.ina.certh.gr/tools/circoletto/) based on BLASTP searches with an E-value of 1e^−1^ threshold [50].

## Figures and Tables

**Table 1:** Number of Illumina reads per alfalfa sample mapped to viral reference sequences.

Sample No.	No. of Contigs >200 bp	Total Reads After QC	AMV			BLRV *	ARaV			dsRNA Viruses			Total Viral Reads	
													No.	%
			RNA1 *	RNA2 *	RNA3 *		RNA1 *	RNA3 *	RNA4 *	MsAV1 *	MsAPV RdRP *	MsAPV CP *		
**1**	273	85,486	12,631	2292	3693	0	0	0	0	182	203	384	19,001	22.2
**2**	245	92,960	10,264	1027	1874	0	45	132	156	81	5209	6318	19,076	20.5
**3**	378	91,818	405	74	108	0	0	29	0	138	186	524	2223	2.4
**4**	551	74,794	296	32	81	0	0	0	0	78	86	287	1619	2.1
**5**	158	77,372	4627	359	697	0	0	9	0	730	497	1095	8129	10.5
**6**	306	97,815	824	56	93	0	0	0	0	6073	144	369	7458	7.6
**7**	146	76,427	1617	84	165	0	0	0	0	299	253	445	2959	3.8
**8**	140	83,257	481	65	118	0	0	0	0	108	210	372	1252	1.5
**9**	156	91,252	2439	149	488	0	34	33	0	1495	2504	2408	7983	8.7
**10**	244	94,339	8705	810	515	146	0	0	0	204	12,419	5495	23,866	25.2

* GenBank accession numbers of virus sequences used for reference mapping: AMV RNA1-3: KC881008-10, X01572 and MF990285, BRLV: KR261610, MsAV1: BK010406, MsAPV1-RdRP: MK292286, MsAPV1-CP: MK292287, wheat mosaic virus (emaravirus): RNA1: KT988869, RNA 3: KT988863 and RNA4: KT970502. AMV: alfalfa mosaic virus, BLRV: bean leafroll virus, ARaV: alfalfa ringspot-associated virus, MsAV1: *Medicago sativa* amalgavirus 1, MsAPV1: *Medicago sativa* alphapartitivirus 1.

**Table 2:** Alfalfa samples used to detect and characterize RNA viruses.

Sample No	Location	Symptom	Detected RNA Viruses and Detection Methods				
			AMV	BLRV	MsAV1 *	MsAPV1 *	ARaV *
1	Victoria	Yellow mosaic	A, B, C	-	B	B	-
2	Victoria	Ringspot, yellow mosaic	A, B, C	-	B, C	B, C	B, C
3	South Australia	Ringspot	B, C	-	B	B	B, C
4	Victoria	Yellow patches, mild ringspot	B, C	-	B	B	-
5	Victoria	Ringspot	A, B, C	-	B	B, C	B
6	South Australia	Ringspot	B, C	-	B, C	B	-
7	Victoria	Reddening	A, B, C	-	B	B, C	-
8	Victoria	Leaf rolling, enation	B, C	-	B	B	-
9	South Australia	Ringspot, yellow patches	B, C	-	B, C	B, C	B, C
10	South Australia	Leaf curling	A, B, C	A, B, C	B	B, C	-

Symptoms are shown in Figure 2. Viruses were detected by A: duplex RT-PCR [45], B: high throughput sequencing (HTS), and C: RT-PCR. (-): not detected. (*) viruses were first identified by HTS and then validated by RT-PCR. AMV: alfalfa mosaic virus, BLRV: bean leaf roll virus, ARaV: alfalfa ringspot-associated virus, MsAV1: *Medicago sativa* amalgavirus 1, MsAPV1: *Medicago sativa* alphapartitivirus 1.

## Supplementary Materials

The following are available online at https://www.mdpi.com/2076-0817/9/3/214/s1, Figure S1: Agarose gel electrophoresis of alfalfa RNA and DNA virus amplicons, Figure S2: RT-PCR verification of HTS-detected ssRNA and dsRNA viruses in total RNA extracts of Australian alfalfa samples, Figure S3: Predicted secondary structure of partial 5′-untranslated regions of ARaV RNA1 compared with WMoV and HPWMoV, Figure S4: Sequence assembly of MsAPV1 dsRNA1 and dsRNA2, and MsAV1 genome sequences from high throughput sequencing data using the Geneious mapper assembly, Figure S5: Predicted secondary structure of 5′ UTR of MsAPV1 dsRNA1, 5′ UTR of MsAV1 and 3′ UTR of MsAV1, Figure S6: Predicted secondary structure of the *Medicago sativa* amalgavirus 1 coat protein, Table S1: Characteristics of viral reads that mapped to alfalfa mosaic virus and bean leaf roll virus, Table S2: Comparison of complete nucleotide sequences of alfalfa mosaic virus isolates, Table S3: Characteristics of viral reads mapped to the genome segments of emaraviruses, Table S4: List of primers designed for RT-PCR and Sanger sequencing to verify dsRNA and ssRNA viral sequences identified by high throughput sequencing, Table S5: Characteristics of viral reads mapped to dsRNA viruses, Table S6: List of Illumina barcodes used to identify individual samples, Table S7: GenBank sequence accession numbers of viruses in the families *Amalgaviridae*, *Partitiviridae* and *Totiviridae* used for pairwise sequence comparisons and to construct phylogenetic trees, Table S8: GenBank accession numbers of viruses in the families *Fimoviridae*, *Tospoviridae* and *Phenuiviridae* used to construct *Bunyavirales* phylogenetic trees, Table S9: GenBank accession numbers for sequences of DNA viruses used to construct phylogenetic tree for geminiviruses, Table S10: GenBank accession numbers of chickpea chlorosis Australia virus isolates used to construct π diversity graph and sequence identity matrix.
