# Supplementary material for: Viromes of Ten Alfalfa Plants in Australia Reveal Diverse Known Viruses and a Novel RNA Virus
Source: Pathogens. 2020 Mar 13;9(3):214. doi: 10.3390/pathogens9030214 (PMC7157637; doi:10.3390/pathogens9030214)
Supplement: Supplementary file 1 [file pathogens-09-00214-s001.pdf]

Supplementary Materials

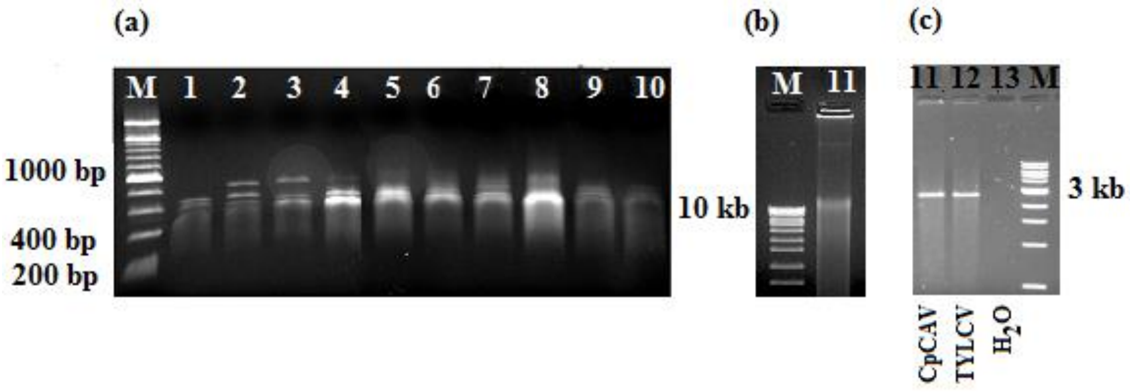

Figure S1. Agarose gel electrophoresis of alfalfa RNA and DNA virus amplicons. (**a**) Amplification and barcoding of cDNA synthesized from dsRNA of ten alfalfa samples: Lanes 1 to 10 correlate with samples 1–10 (Figure 2) and M is O'RangeRuler™ 200 bp DNA ladder, (**b**) Rolling circle amplification products of total DNA derived from sample 11, M is MassRuler High Range DNA 10 kb ladder. (**c**) *Eco*RI digest of rolling circle amplified total DNA, lane 11: sample 11, infected with chickpea chlorosis Australia virus (CpCAV), lane 12: tomato yellow leaf curl virus (TYLCV) positive control and lane 13: nuclease-free water. M is NEB Quick-Load® 1 kb DNA Ladder. Size of selected DNA size markers are indicated in the margins.

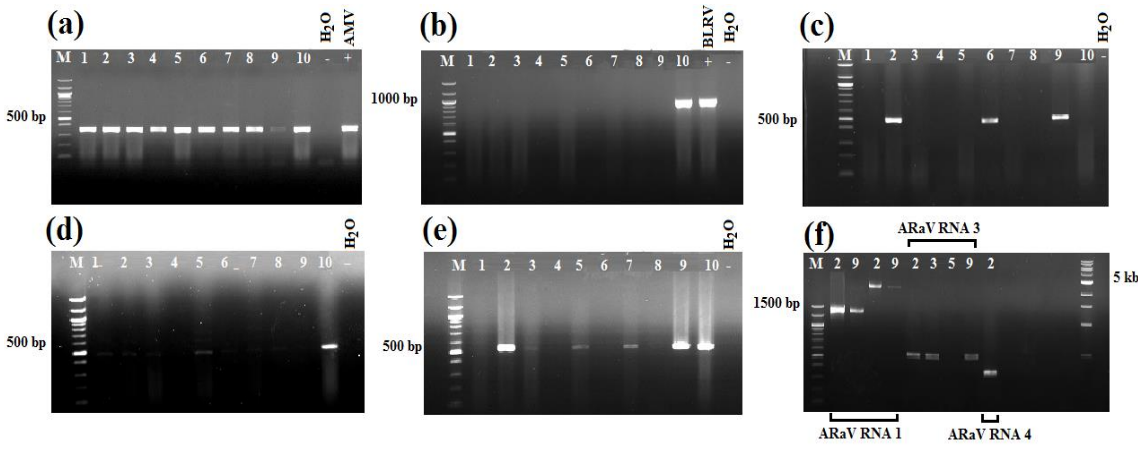

Figure S2. RT-PCR verification of HTS-detected ssRNA and dsRNA viruses in total RNA extracts of Australian alfalfa samples. RT-PCR to amplify (**a**) AMV-CP (**b**) BLRV-CP (**c**) MsAV1-CP (**d**) MsAPV1-CP (**e**) MsAPV1-RdRP specific products and (**f**) RT-PCR results with ARaV RNAs 1, 2 and 3. Specific primer sets are listed in Table S4. Samples 1–10 correspond to the leaf samples shown in Figure 2. Amplicons were electrophoresed in 1% agarose/Tris-borate-EDTA. Sizes of selected DNA markers are indicated in the margins. AMV: alfalfa mosaic virus, BLRV: bean leafroll virus, ARaV: alfalfa ringspot-associated virus, MsAV1: *Medicago sativa* amalgavirus 1, MsAPV1: *Medicago sativa* alphapartitivirus 1.

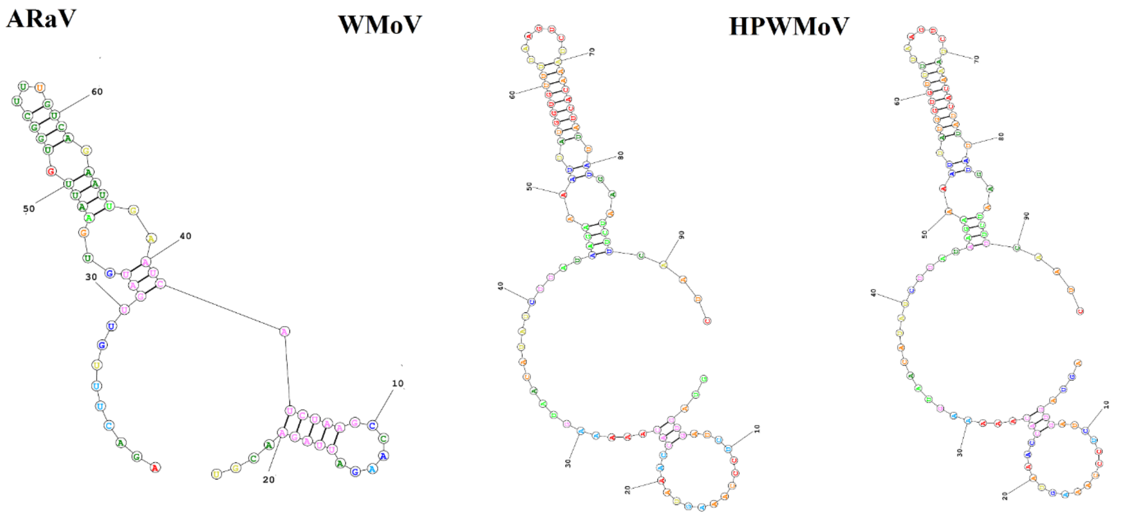

Figure S3. Predicted secondary structure of partial 5'-untranslated regions of alfalfa ringspot-associated virus (ARaV, MK648430) RNA1 compared with wheat mosaic virus (WMoV, KT988860) and High Plains wheat mosaic virus (HPWMoV, KJ939623).

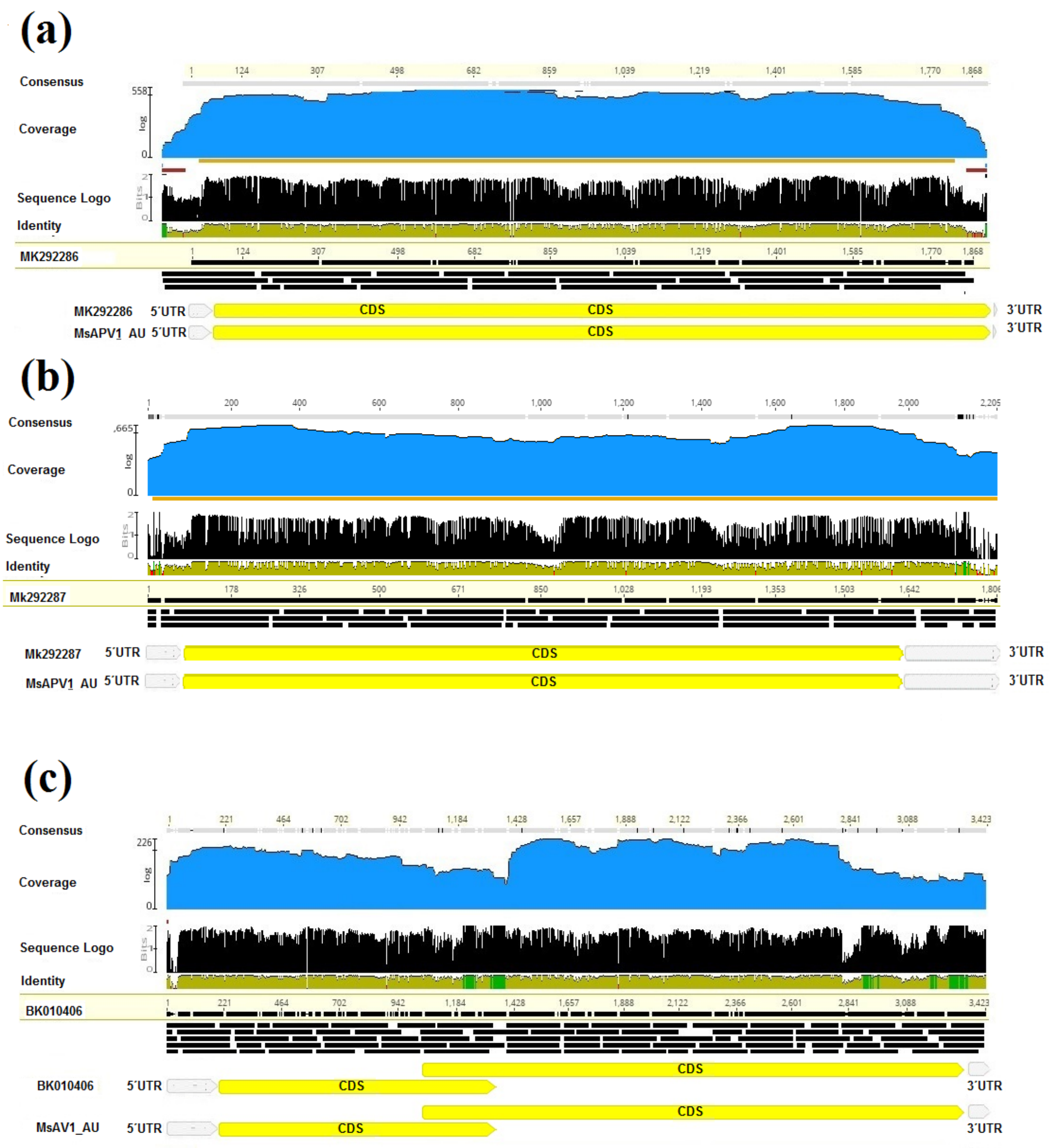

Figure S4. Sequence assembly of (**a**) *Medicago sativa* alphapartitvirus 1 dsRNA 1 (MsAPV1-dsRNA 1), (**b**) MsAPV1-dsRNA 2) and (**c**) *Medicago sativa* amalgavirus 1 (MsAV1) genome sequences from high throughput sequencing data using the Geneious mapper assembly. Tracks from top to bottom of graph represent coverage per base and sequence identity from red (low) to green (high). Coding regions (CDS) and UTR are shown for the reference and assembled Australian sequences.

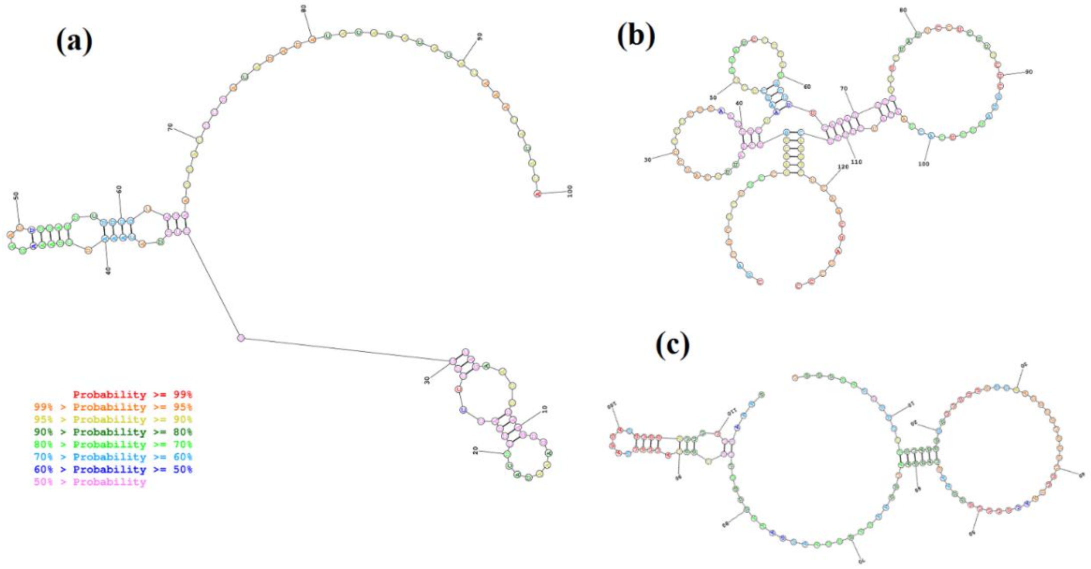

Figure S5. Predicted secondary structure of (**a**) 5'-untranslated region (5′UTR) of *Medicago sativa* alphapartitivirus 1 dsRNA1, (**b**) 5′UTR of *Medicago sativa* amalgavirus 1 (MsAV1) and (**c**) 3′UTR of MsAV1.

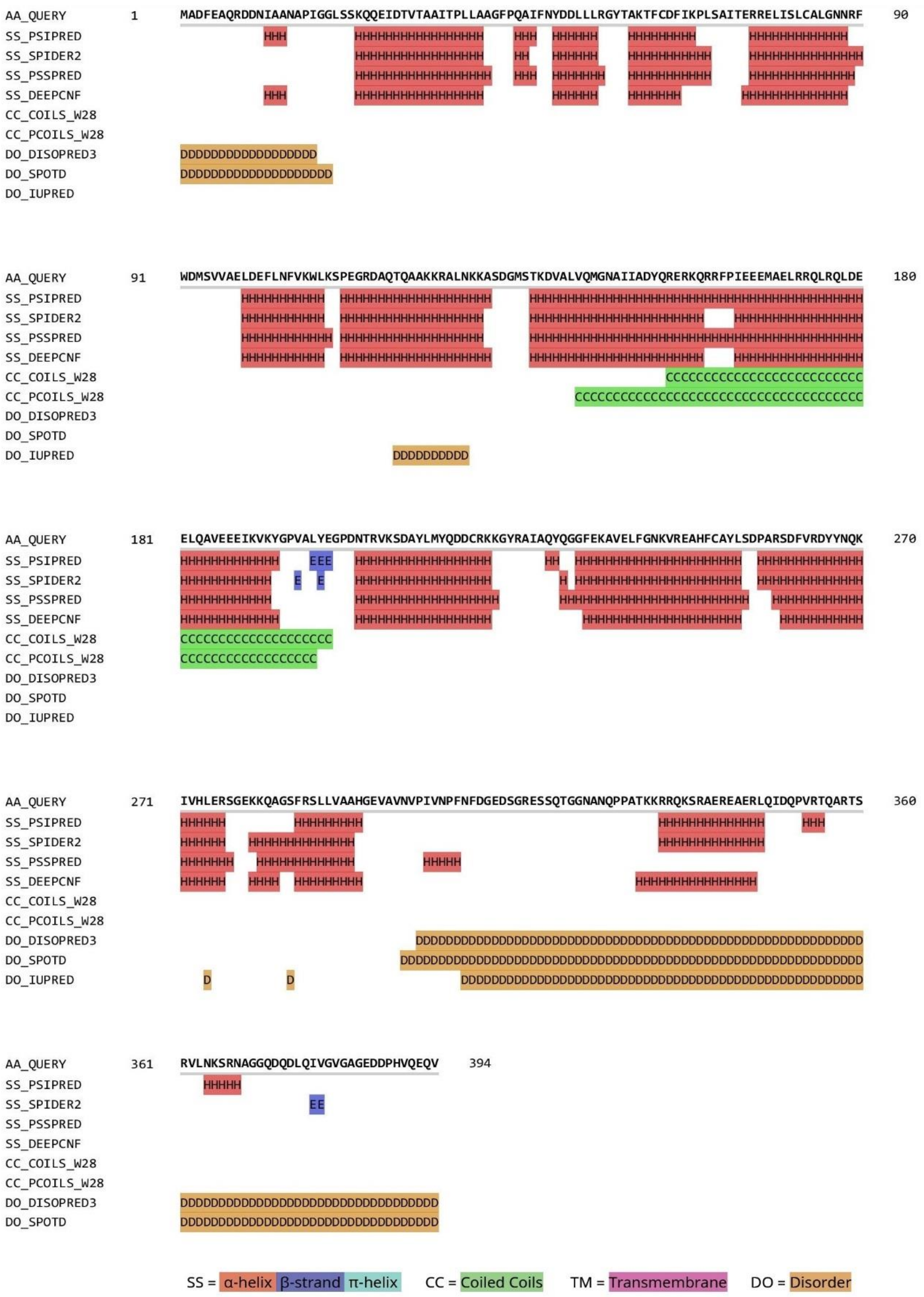

Figure S6. Predicted secondary structure of the *Medicago sativa* amalgavirus 1 coat protein. The protein structural domains were predicted with Emboss online software (http://www.bioinformatics.nl/cgi-bin/emboss/garnier) using default parameters.

Table S1. Characteristics of viral reads that mapped to alfalfa mosaic virus and bean leaf roll virus.

Sample No	Total No of Reads	AMV genome segments/BLRV	No. Mapped Reads	Proportion of Total Viral Reads %	Fold Coverage	Completeness of RefSeq %
**1**	85,486	RNA 1	12,631	14.7	2871	99.5
		RNA 2	2292	2.6	784	99.8
		RNA 3	3693	4.3	517	99.9
**2**	92,960	RNA 1	10,264	11	1573	100
		RNA 2	1027	1.1	176	99.9
		RNA 3	1874	2	623	96.8
**3**	91,818	RNA 1	405	0.4	77	99.7
		RNA 2	74	0.08	16	96.1
		RNA 3	108	0.1	29	98.9
**4**	74,794	RNA 1	296	0.4	51	98.8
		RNA 2	32	0.4	7	82.7
		RNA 3	81	0.1	20	100
**5**	77,372	RNA 1	4627	5.9	1132	100
		RNA 2	359	0.5	68	98.2
		RNA 3	697	0.9	243	94.4
**6**	97,815	RNA 1	824	0.8	208	99.9
		RNA 2	56	0.05	11	80.4
		RNA 3	93	0.09	21	100
**7**	76,427	RNA 1	1617	2.1	622	99.2
		RNA 2	84	0.1	25	92.6
		RNA 3	165	0.2	64	100
**8**	83,257	RNA 1	481	0.5	90	99.1
		RNA 2	65	0.07	13	95.6
		RNA 3	118	0.1	29	92.1
**9**	91,252	RNA 1	2439	2.6	836	100
		RNA 2	149	0.1	42	97
		RNA 3	488	0.5	228	90.1
**10**	94,339	RNA 1	8705	9.2	1681	100
		RNA 2	810	0.8	226	95.7
		RNA 3	515	0.5	116	100
		BLRV	146	0.15	73	36.7

Table S2. Comparison of complete nucleotide sequences of alfalfa mosaic virus isolates.

**Isolate Origin**	**RNA 1**		**P1 Gene**					
	**nt**	**Identity%**	**nt**	**nt %**	**aa %**			
Australia (MK648424)	3643	-	3381	-	-			
Australia (LC485018)	3753	**99.1***	3381	**99.1**	**99.9**			
Argentina (KC881008)	3643	**99.2**	3381	**99.1**	**99.8**			
China (HQ316635)	3643	98.7	3381	98.7	99.5			
Italy (FN667965)	3643	98.1	3381	98.2	98.7			
Spain (FR715040)	3643	96.8	3381	96.7	98.5			
USA (L00163)	3644	97.6	3381	97.7	98.6			
Canada (MF990284)	3631	98.1	3381	98.2	99.6			
UK (KY810767)	3643	96.6	3381	97.0	98.4			
**Isolate Origin**	**RNA 2**		**P2 gene**					
	**nt**	**nt%**	**nt**	**nt %**	**aa %**			
Australia (MK648425)	2592	-	2373	-	-			
Australia (LC485016)	2579	94.7	2373	94.8	95.2			
Argentina (KC881009)	2593	95.7	2373	95.8	95.2			
China (HQ316636)	2595	97.5	2373	97.8	98.4			
Italy (FN667966)	2593	95.2	2373	95.4	94.8			
Spain (FR715041)	2594	95.2	2373	95.2	94.7			
USA (X01572)	2593	**97.8**	2373	**98.1**	96.5			
Canada (MF990285)	2497	**97.8**	2373	97.9	**98.4**			
UK (KY810768)	2593	95.6	2373	95.7	94.8			
**Isolate Origin**	**RNA 3**		**MP gene**			**CP gene**		
	**nt**	**nt%**	**nt**	**nt%**	**aa%**	**nt**	**nt%**	**aa%**
Australia (MK648426)	2038	-	903	-	-	666	-	-
Australia (LC485017)	2033	99.3	903	99.1	99.3	666	**99.7**	99.5
Argentina (KC881010)	2038	**99.4**	903	**99.6**	**99.7**	657	99.2	**100**
China (HQ316637)	2041	97.4	903	97.1	98.7	657	97.6	98.6
Italy (FN667967)	2038	97.4	903	97	97	657	97.1	98.6
Spain (FR715042)	2037	95.5	903	94.6	96.3	666	95.2	95.9
USA (K03542)	2142	93.2	903	97.7	97	666	98.2	97.3
Canada (MF990286)	2041	99.2	903	99.1	99.3	666	99.7	100
UK (KY810769)	2039	95.2	903	94.2	95.7	666	94.9	94.6

* Highest sequence identity is shown in bold. MP=movement protein, CP=coat protein, UK=United Kingdom, USA=United States of America.

Table S3. Characteristics of viral reads mapped to the genome segments of emaraviruses.

Sample No	Total No of Reads	Emaravirus	Mapped Reads	Proportion of Total Viral Reads %	Fold Coverage	Completeness of RefSeq %
**2**	92,960	RNA1	45	0.04	11	5.9
**2**	92,960	RNA3	132	0.14	61	12.6
**2**	92,960	RNA4	156	0.16	95	13.7
**3**	91,818	RNA3	29	0.03	15	1.3
**5**	77,372	RNA3	9	0.01	4	1.5
**9**	91,252	RNA1	34	0.03	10	3.5
**9**	91,252	RNA3	33	0.03	19	13.3

Table S4. List of primers designed for RT-PCR and Sanger sequencing to verify dsRNA and ssRNA virus sequences identified by high throughput sequencing.

Target Sequence	Primer Name	Sequence (5′ – 3′)	Product Size (bp)
**MsAPV1-RdRP**	Partitivirus RdRP 415 F	TTTCTGACTTGCGCCATTAC	551
	Partitivirus RdRp 965 R	CCAGTCGAGAGTAGCAACAA	
**MsAPV1-CP**	Partitivirus CP 941 F	GTCACCACCAATTCCCAATTT	500
	Partitivirus CP1,440 R	GTTGTAGTAGGGATGGGTCG	
**MsAV1-CP**	Amalgavirus 301 F	GTTACACCGCCAAGACTTTC	500
	Amalgavirus 803 R	ACCTTTCTTCCGACAATCATC	
**ARaV/RNA 1**	Emara RNA1 N87 F	TTTCTAGGTGTTGGGTAATCAA	1500
	Emara RNA1 N124/85 R	GTGATGTGACATATGATTCACC	
**ARaV/RNA 1**	Emara RNA1 N87 F	TTTCTAGGTGTTGGGTAATCAA	3000
	Emara RNA1 N8/46 R	ACGTTCTAATCTTGGCTTAGAT	
**ARaV/RNA 1**	Emara RNA 1 1041 F	ATCAGGGAATGCTTTATCATCT	*
	Emara RNA 1 629 F	TTACCAGAAGAAGCTATCAGTC	*
**ARaV/RNA 3**	Emara RNA3 N7 F	TTAACAACAAATCGTCCAAAAC	500
	Emara RNA3 N7 R	TAGATGGTCCCAACTTGTTAAA	
**ARaV/RNA 4**	Emara RNA4 N77 F	GTATCACATATTTCTGTGCTGG	360
	Emara RNA4 N77 R	TAATTCATTACAAGCCACACAC	
**BLRV-CP**	BLRV CP-F1	TAGGTTCCTTCGATTACAAG	955
	BLRV CP-R1	CTTCAATATTCGTCCAGTTC	
**AMV-CP**	AMV CP 214F	GCGAGATTCCTCTACAGTTT	363
	AMV CP 576R	GACCCAAACTTCGTTGAATC	

* Primers were used for sequencing only.

Table S5. Characteristics of viral reads mapped to dsRNAs of *Medicago sativa* amalgavirus 1, *Medicago sativa* alphapartitivirus 1 dsRNA1 and *Medicago sativa* alphapartitivirus 1 dsRNA2.

Sample	Total No of Reads	Detected Viral RNAs	Mapped Reads	Proportion of Total Viral Reads %	Fold Coverage	Completeness of RefSeq %
**1**	85,486	MsAV1	182	0.2	53	95.3
		MsAPV1 RNA1	203	0.2	49	98.8
		MsAPV1 RNA2	384	0.4	16	100
**2**	92,960	MsAV1	81	0.08	18	83.3
		MsAPV1 RNA1	5209	5.6	1070	92.9
		MsAPV1 RNA2	6318	6.7	737	100
**3**	91,818	MsAV1	138	0.1	18	95.2
		MsAPV1 RNA1	186	0.2	43	99.1
		MsAPV1 RNA2	524	0.6	39	100
**4**	74,794	MsAV1	78	0.1	8	93.2
		MsAPV1 RNA1	86	0.1	18	96.7
		MsAPV1 RNA2	287	0.4	19	99
**5**	77,372	MsAV1	730	0.9	216	100
		MsAPV1 RNA1	497	0.6	138	96.1
		MsAPV1 RNA2	1095	1.4	71	100
**6**	97,815	MsAV1	6073	6.2	836	100
		MsAPV1 RNA1	144	0.1	30	96.6
		MsAPV1 RNA2	369	0.4	27	100
**7**	76,427	MsAV1	299	0.4	80	98.6
		MsAPV1 RNA1	253	0.3	56	99.8
		MsAPV1 RNA2	445	0.6	31	98
**8**	83,257	MsAV1	108	0.1	16	97.8
		MsAPV1 RNA1	210	0.2	38	100
		MsAPV1 RNA2	372	0.4	24	99
**9**	91,252	MsAV1	1495	1.6	226	100
		MsAPV1 RNA1	2504	2.7	558	100
		MsAPV1 RNA2	2408	2.6	235	100
**10**	94,339	MsAV1	204	0.2	49	98.7
		MsAPV1 RNA1	12,419	13	2657	100
		MsAPV1 RNA2	5495	5.8	555	100

Table S6. List of Illumina barcodes used to identify individual samples.

Barcode No.	Sequence (5′ to 3′)	Annealing temp (° C)	Barcoded Sample No.
1	ATCACGCCTTCGGATCCTCC	58.0	1
2	CGATGTCCTTCGGATCCTCC	58.0	2
3	TTAGGCCCTTCGGATCCTCC	58.3	3
4	TGACCACCTTCGGATCCTCC	58.3	4
5	ACATGTCCTTCGGATCCTCC	57.5	5
6	CAGATCCCTTCGGATCCTCC	57.3	6
7	ACTTGACCTTCGGATCCTCC	57.5	7
8	GATCAGCCTTCGGATCCTCC	57.3	8
9	GGCTAGCCTTCGGATCCTCC	58.0	9
10	CTTGTACCTTCGGATCCTCC	55.0	10

Table S7. GenBank sequence accession numbers of viruses in the families *Amalgaviridae, Partitiviridae* and *Totiviridae* used for pairwise sequence comparisons and to construct phylogenetic trees.

**Family:** ***Amalgaviridae***		
**Genus:** ***Amalgavirus***		
**Virus name**	**Abbrev**	**Accession #**
Allium cepa amalgavirus 1	AcAV1	BK010347
Allium cepa amalgavirus 2	AcAV2	BK010348
Anthoxanthum odoratum amalgavirus 1	AoAV1	BK010356
Camellia oleifera amalgavirus 1	CoAV1	BK010409
Capsicum annuum amalgavirus 1	CaAV1	BK010407
Cistus incanus RNA virus 1	CiRV1	MG833407
Cleome droserifolia amalgavirus 1	CdAV1	BK010408
Cucumis melo amalgavirus 1	CmAV1	QBC66123
Erigeron breviscapus amalgavirus 1	EbAV1	BK010410
Erigeron breviscapus amalgavirus 2	EbAV2	BK010411
Festuca pratensis amalgavirus 1	FpAV1	BK010412
Festuca pratensis amalgavirus 2	FpAV2	BK010413
Festuca pratensis amalgavirus 3	FpAV3	BK010350
Gevuina avellana amalgavirus 1	GaAV1	BK010351
Lolium perenne amalgavirus 1	LpAV1	BK010352
Medicago sativa **amalgavirus 1**	**MsAV1_AU**	**MK648427**
Medicago sativa amalgavirus 1	MsAV1	BK010406
Neurachne minor latent virus	NmLV	MF325033
Pinus patula amalgavirus 1	PpAV1	BK010353
Rhododendron virus A	RHV-A	HQ128706
Rubber dandelion latent virus 1	RdLV1	MF197380
Rubber dandelion latent virus 2	RdLV2	MF197379
Salvia hispanica RNA virus 1	ShRV1	MH988691
Secale cereale amalgavirus 1	ScAV1	BK010349
Southern tomato virus	STV	NC_011591
Spinach amalgavirus 1	SpAV1	KY695011
Vicia cryptic virus M	VCV-M	EU371896
Zostera marina amalgavirus 1	ZmAV1	KY783316
Zostera marina amalgavirus 2	ZmAV2	KY783317
Zygosaccharomyces bailii virus Z*	ZbvZ	KU200450

Table S7: (continued)

**Family:** ***Partitiviridae***
**Genus:** ***Alphapartitivirus***

Virus name	Abbrev	Accession #
Arabidopsis halleri partitivirus 1	AhPV1	LC151461 / LC151462
Beet cryptic virus 1	BCV1	EU489061 / EU489062
Carrot cryptic virus segment RNA 1	CCV	FJ550604 / FJ550605
***Medicago sativa* alphapartitivirus 1**	**MsAPV1_AU**	**MK648428 / MN660231**
Medicago sativa alphapartitivirus 1	MsAPV1	MF443256 / MF443257
Medicago sativa alphapartitivirus 1	MsAPV1	MK292286 / MK292287
Medicago sativa alphapartitivirus 1	MsAPV1	MH846126 / MH846125
Medicago sativa alphapartitivirus 2	MsAPV2	MK292288 / MK292289
Raphanus sativus cryptic virus 1	RsCV1	MH427289 / MH427288
Red clover cryptic virus 1	RCCV1	KF484724 / KF484725
Rose partitivirus	RoPV	KU896858 / KU896859
Vicia cryptic virus	VCV	AY751737 / AY751738
White clover cryptic virus 1	WCCV1	AY705784 / AY705785
**Genus:** ***Betapartitivirus***		
**Virus name**	**Abbrev**	**Accession #**
Cannabis cryptic virus	CanCV	JN196536 / JN196537
Crimson clover cryptic virus 2	CCCV2	JX971982 / JX971983
Dill cryptic virus 2	DCV2	JX971984 / JX971985
Grapevine partitivirus	GPV	JX658566 / NA
Hop trefoil cryptic virus 2	HTCV2	JX971980 / JX971981
Red clover cryptic virus 2	RCCV2	JX971978 / JX971979
White clover cryptic virus 2	WCCV2	JX971976 / JX971977
**Genus:** ***Deltapartitivirus***		
**Virus name**	**Abbrev**	**Accession #**
Citrullus lanatus cryptic virus	CiLCV	KC429582 / KC429583
Fig cryptic virus	FCV	FR687854 / FR687855
Medicago sativa deltapartitivirus 1	MsDPV1	MF443258 / MF443259
Pepper cryptic virus 1	PepCV1	JN117276 / JN117277
Persimmon cryptic virus	PeCV	HE805113 / HE805114
Totiviridae **Family**		
**Genus:** ***Totivirus***		
**Virus name**	**Abbrev**	**Accession #**
Red clover powdery mildew-associated totivirus*	RPaTV	LC075486 / LC075486

* Virus used as an outgroup to root NJ tree of partitiviruses and amalgaviruses, respectively. NA: Not available Bold text: virus sequences detection in this study.

Table S8. GenBank accession numbers of viruses in the families *Fimoviridae, Tospoviridae* and *Phenuiviridae* used to construct *Bunyavirales* phylogenetic trees.

Virus name	Abbrev	Accession # [a]
**Family: *Fimoviridae***		
**Genus: *Emaravirus***		
**Virus name**	**Abbrev**	**Accession # [a]**
Actinidia chlorotic ringspot-associated virus	AcCRaV	KT861483 / KT861481
**Alfalfa ringspot-associated virus**	**ARaV**	**MK648429 / MK648430**
European mountain ash ringspot-associated virus	EMARAV	KJ439588 /AY563040
Fig mosaic virus	FMV	FM991954 /AM941711
Pistacia virus B	PiVB	MH727574 / MH727572
Pigeonpea sterility mosaic virus 1	PPSMV1	MH374922 / MH374920
Pigeonpea sterility mosaic virus 2	PPSMV2	MH374927 / MH374925
Raspberry leaf blotch virus	RLBV	FR823301 / FR823299
Rose rosette virus	RRV	HQ891896 / MH581220
Redbud yellow ringspot-associated emaravirus	RYRSaV	JF795547 / JF795479
Tomato chlorotic spot virus	TCSV	KX463274 / KX463272
Wheat mosaic virus	WmoV	KT988862 / KT988860
**Family: *Tospoviridae***		
**Genus: *Orthotospovirus***		
**Virus name**	**Abbrev**	**Accession # [a]**
Impatiens necrotic spot virus	INSV	MH171174 / MH171172
Tomato chlorotic spot virus	TCSV	KX463274 / KX463272
Tomato spotted wilt virus	TSWV	AY848922 / KJ575620
Zucchini lethal chlorosis virus	ZLCV	KU641380 / KU641378
**Family: *Phenuiviridae***		
**Genus: Tenuivirus**		
**Virus name**	**Abbrev**	**Accession # [a]**
Rice grassy stunt virus*	RGSV	AF397468 / AB032180

* Used as an outgroup to root the NJ tree. Bold text: virus isolate sequenced in this study, [a] Genbank accession numbers for nucleocapsid/RNA-dependent RNA polymerase amino acid sequences, respectively.

Table S9. GenBank accession numbers for sequences of DNA viruses used to construct phylogenetic tree for geminiviruses.

Family: *Geminiviridae*		
Genus: *Mastrevirus*		
**Virus name**	**Abbrev**	**Accession # [a]**
Axonopus compressus streak virus	ACSV	KJ437671
Bromus catharticus striate mosaic virus	BCSMV	HQ113104
**Chickpea chlorosis Australia virus isolate SA**	**CpCAV**	**MK648431**
Chickpea chlorosis Australia virus	CpCAV	JN989422
Chickpea chlorotic dwarf virus	CpCDV	DQ458791
Chickpea chlorosis virus-A	CpCV/A	GU256530
Chickpea redleaf virus	CpRLV	GU256532
Chickpea yellow dwarf virus	CpYDV	KM377674
Chickpea yellows mastrevirus	CpYV	JN989439
Chloris striate mosaic virus	CSMV	M20021
Digitaria didactyla striate mosaic virus	DDSMV	HM122238
Maize streak virus	MSV	AF003952
Wheat dwarf virus	WDV	EF536860
**Genus: *Capulavirus***		
**Species**	**Abbrev**	**Accession # [a]**
Alfalfa leaf curl virus	ALCV	KX574859
Euphorbia caput-medusae latent virus	EcmLV	KT214386
French bean severe leaf curl virus	FbSLCV	JX094280
Plantago lanceolata latent virus	PlLV	KT214389
**Genus: *Topocuvirus***		
**Species**	**Abbrev**	**Accession # [a]**
Tomato pseudo-curly top virus*	TPCTV	X84735

*Virus used as an outgroup. Bold text: virus sequence determined in this study.

Table S10. GenBank accession numbers of chickpea chlorosis Australia virus isolates used to construct π diversity graph and sequence identity matrix

No	Accession No	Host	Host Common Name	Date	Isolate No
**1**	JN989419	*Cicer arietinum*	Chickpea	2010	2612
**2**	JN989420	*C. arietinum*	Chickpea	2010	2614
**3**	JN989421	*C. arietinum*	Chickpea	2010	2614a
**4**	JN989422	*C. arietinum*	Chickpea	2002	3494I
**5**	JN989423	*C. arietinum*	Chickpea	2003	3768F
**6**	KC172686	*C. arietinum*	Chickpea	2003	3758E
**7**	KC172687	*C. arietinum*	Chickpea	2003	3768C
**8**	KC172688	*C. arietinum*	Chickpea	2003	3773G
**9**	KC172689	*C. arietinum*	Chickpea	2002	3460E
**10**	KC172691	*C. arietinum*	Chickpea	2011	21
**11**	KC172692	*C. arietinum*	Chickpea	2011	41
**12**	KC172693	*C. arietinum*	Chickpea	2011	34
**13**	**MK648431**	***Medicago sativa***	**Alfalfa**	**2016**	**SA9**
**14**	JN989418	*Phaseolus vulgaris*	Bean	2007	2008

Bold text: Virus sequence determined in this study.
